# Molecular characterization and predictors of relapse in patients with Ph + ALL after frontline ponatinib and blinatumomab

**DOI:** 10.1186/s13045-025-01709-y

**Published:** 2025-05-14

**Authors:** Nicholas J. Short, Hagop Kantarjian, Ken Furudate, Nitin Jain, Farhad Ravandi, Omer Karrar, Sanam Loghavi, Lewis Nasr, Fadi G. Haddad, Jayastu Senapati, Rebecca Garris, Koichi Takahashi, Elias Jabbour

**Affiliations:** 1 Department of Leukemia, The University of Texas MD Anderson Cancer Center, Unit 428, 1515 Holcombe Boulevard, Houston, TX 77030 USA; 2 Department of Hematopathology, The University of Texas MD Anderson Cancer Center, Houston, TX USA

**Keywords:** Blinatumomab, Ponatinib, Philadelphia chromosome, Relapse

## Abstract

**Background:**

Several studies have suggested that chemotherapy-free regimens consisting of blinatumomab and a BCR::ABL1 tyrosine kinase inhibitor are highly effective in Philadelphia chromosome-positive acute lymphoblastic leukemia (Ph + ALL). However, the clinical and molecular characteristics that predict for relapse with these chemotherapy-free regimens are largely unknown.

**Methods:**

We conducted a prospective phase II clinical trial of the combination of blinatumomab and ponatinib in 76 patients with newly diagnosed Ph + ALL. Patients received 12–15 doses of intrathecal chemotherapy as central nervous systemic (CNS) prophylaxis. The patterns of relapse and the clinical and molecular predictors of relapse were analyzed.

**Results:**

With a median follow-up of 29 months, the estimated 3-year event-free survival rate was 78% and the 3-year overall survival rate was 88%. Ten patients (13%) relapsed, with a median time to relapse of 18 months (range, 8–24 months). Six relapses occurred only in extramedullary sites (CNS, *n* = 5; peritoneum and lymph nodes, *n* = 1). CD19 expression remained high at relapse in all patients. On univariate analysis, factors associated with an increased risk of relapse were: white blood cell (WBC) ≥ 70 × 10^9^/L at diagnosis (sHR 8.86 [95% CI 2.33–33.70]; *P* = 0.001), CNS involvement at diagnosis (sHR 6.87 [95% CI 1.54–30.68]; *P* = 0.01), and *VPREB1* deletion (sHR 4.06 [95% CI 1.05–15.76]; *P* = 0.04). WBC ≥ 70 × 10^9^/L was present in 22% of the cohort and was associated with a 53% cumulative incidence of relapse (CIR), as compared with a CIR rate of 6% for patients with WBC < 70 × 10^9^/L. Neither *IKZF1*^plus^ genotype, *BCR::ABL1* transcript type, nor measurable residual disease kinetics by next-generation sequencing for IG/TR rearrangements significantly impacted the risk of relapse. High WBC at diagnosis was the only variable significantly associated with relapse on multivariate analysis (sHR 16.29 [95% CI 2.35–113.00; *P* = 0.005).

**Conclusions:**

WBC ≥ 70 × 10^9^/L is a high-risk feature in patients with Ph + ALL receiving frontline blinatumomab and ponatinib and may supersede the prognostic importance of baseline molecular features. Alternative frontline treatment strategies may be needed for these patients to reduce the risk of relapse and improve long-term outcomes.

**Trial registration:**

ClinicalTrials.gov (NCT03263572).

**Supplementary Information:**

The online version contains supplementary material available at 10.1186/s13045-025-01709-y.

## Introduction

The historical standard of care for adults with newly diagnosed Philadelphia chromosome-positive (Ph+) acute lymphoblastic leukemia (ALL) was chemotherapy plus a BCR::ABL1 tyrosine kinase inhibitor (TKI), followed by allogeneic stem cell transplant (alloSCT) in first remission [1]. Recently, chemotherapy-free regimens combining blinatumomab with a BCR::ABL1 TKI have shown high rates of deep molecular responses and encouraging survival in Ph + ALL, even without routine alloSCT consoildation [2, 3]. In the D-ALBA study, which evaluated the combination of blinatumomab and dasatinib in newly diagnosed Ph + ALL, the estimated 4-year disease-free survival (DFS) and overall survival (OS) rates were 76% and 81%, respectively [2]. Encouraging outcomes have also been observed with frontline use of blinatumomab and ponatinib [3, 4]. In a recent update, a 3-year OS rate of 91% was observed, despite < 5% of patients undergoing alloSCT in first remission [3]. However, despite promising outcomes in multiple studies of frontline blinatumomab + TKI combinations, some patients still relapse, and a relatively high proportion of these relapses occur in extramedullary spaces, particularly in the central nervous system (CNS).

In the D-ALBA study, lack of early molecular response or presence of the *IKZF1*^plus^ genotype (defined as *IKZF1* deletion plus *CDKN2A/B* and/or *PAX5* deletion) were associated with worse DFS [2]. However, the clinical and molecular predictors of relapse with the blinatumomab and ponatinib regimen have not been well-described. Herein, we describe the molecular characteristics and predictors of relapse in patients treated prospectively with blinatumomab and ponatinib for newly diagnosed Ph + ALL.

## Methods

### Patients and study design

This was a single-center, phase 2 study to assess the efficacy and safety of the combination of ponatinib and blinatumomab in patients with newly diagnosed Ph + ALL (ClinicalTrials.gov identifier NCT03263572). Following initial ramp-up (9 mcg/day on days 1–4) in cycle 1, patients received blinatumomab at 28 mcg/day for up to 5 cycles on a standard 4-week on, 2-week off schedule. Ponatinib was given at a dose of 30 mg orally daily until complete molecular response (CMR) was achieved, after which the dose was reduced to 15 mg orally daily and continued for at least 5 years. Initially, patients received 12 doses of intrathecal chemotherapy with alternating doses of cytarabine and methotrexate. However, beginning with patient #64, the number of doses of IT chemotherapy was increased to 15 due to concern for an increased risk of CNS relapse with chemotherapy-free regimens in Ph + ALL. The study specifics, including patient eligibility, detailed treatment plan, response endpoints, and statistical analysis have been previously reported and are detailed in the **Supplemental File** [3, 4]. The study continues to enroll patients.

### Targeted mutation sequencing and single-nucleotide polymorphism (SNP) microarrays

Mutational analysis was prospectively performed at diagnosis using an 81-gene next-generation sequencing panel as previously described, with a sensitivity of 1% (Supplemental Table 1) [5, 6]. Genome-wide copy-number analysis was retrospectively performed on banked samples using SNP microarrays (Illumina Infinium Global Diversity Array-8 v1.0). The raw intensity data (*.idat files) were analyzed using the Genotyping Module in the Illumina GenomeStudio software program version 2.0.3 (Illumina). After correcting for over-segmentation, statistically significant regions (false discovery rate [FDR] < 0.05) with copy-number alterations were identified using the GISTIC 2.0 algorithm [7]. The actual copy-number alterations were manually inspected using conumee R package (version 1.9.0) [8].

### Response and outcomes definitions

CMR was defined as absence of a detectable *BCR::ABL1* transcript by polymerase chain reaction at a sensitivity of 0.01%. Next-generation sequencing (NGS) MRD assessment of IG/TR gene rearrangements was performed on bone marrow specimens using the clonoSEQ assay (Adaptive Biotechnologies) with a sensitivity of 0.0001%. Cumulative incidence of relapse (CIR) was calculated considering death as a competing event. Event-free survival (EFS) was defined as the time from the start of therapy to time of no response, relapse, or death from any cause. Overall survival (OS) was defined as the time from the start of therapy to death from any cause.

### Statistical analysis

This is an interim analysis of the clinical data from this ongoing study, with the primary objective of identifying predictors of relapse based on retrospectively analyzed molecular data. The cutoff for WBC was determined using a time-dependent receiver operating characteristic analysis to determine the optimal WBC threshold for prediction of relapse risk. The Youden Index was applied at each time point to identify the best threshold. We also applied the bootstrap method to confirm the robustness of our findings. For univariate analysis, the variables were pre-selected based on biological considerations and previous publications. Each predictor was examined individually using the Fine-Gray competing risks model, and *P*-values were adjusted for multiple testing using the false discovery rate (FDR) method. *P*-values in cumulative incidence plots are derived from Gray’s test, assessing overall differences in cumulative incidence, while *P*-values in the Forest plot are based on the Fine and Gray model for univariate risk analysis. For multivariate analysis (MVA), we used the Fine-Gray competing risks model, including all statistically significant variables (*P* < 0.05) from the univariate analysis, as well as *IKZF1*^plus^ genotype, given its prior reported association with outcomes in some Ph + ALL studies. Results were presented as subdistribution hazard ratios (sHR) with 95% confidence intervals (CI) in forest plots. These analyses were performed using the cmprsk package (version 2.2.11) in R software (version 4.1.2).

## Results

### Clinical patient characteristics

Between June 2018 and May 2024, 76 patients were treated. The baseline characteristics of the study population are shown in Table 1. The median age of the study population was 50 years (range, 18–83 years), and 28 patients (27%) were ≥ 60 years. The median WBC at diagnosis was 15.4 × 10^9^/L (range, 0.6-322.1 × 10^9^/L). Three patients (4%) had known CNS involvement prior to enrollment. 80% of patients had the p190 BCR::ABL1 transcript, and 20% had the p210 transcript.

**Table 1:** Baseline characteristics and response rates of the study population (*n* = 76)

Baseline characteristics	n/N (%) / median [range]
Age (years)	50 [18–83]
*Age ≥ 60 years*	28 (37)
Baseline cardiovascular risk factor^1^	40 (53)
WBC at diagnosis (x10^9^/L)	15.4 [0.6-322.1]
*WBC at diagnosis ≥ 70 x10*^*9*^*/L*	17 (22)
Central nervous system involvement at diagnosis	3 (4)
CD19 expression (%)	99.8 [74.9–100]
Additional chromosomal abnormalities	37/52 (71)
*BCR::ABL1* transcript	
*p190*	60/75 (80)
*p210*	15/75 (20)
Gene mutations	
*IKZF1*	5/57 (9)
*ASXL1*	3/57 (5)
*DNMT3A*	3/57 (5)
*TET2*	3/57 (5)
*BCORL1*	1/57 (2)
*ETV6*	1/57 (2)
*RUNX1*	1/57 (2)
*SF3B1*	1/57 (2)
Gene deletions	
*IKZF1*	35/48 (73)
*CDKN2A/B*	27/48 (56)
*PAX5*	13/48 (27)
*XBP1*	13/48 (27)
*RB1*	11/48 (23)
*BTG1*	7/48 (15)
*VPREB1*	7/48 (15)
*CD200*	5/48 (10)
*IZKF1*^plus^ genotype^2^	25/48 (52)
**Response rates**	**n/N (%)**
Hematologic response^3^	
*CR*	51/53 (96)
*CRi*	1/53 (2)
*Early death*	1/53 (2)
MRD response^4^	
*CMR after cycle 1*	41/69 (59)
*CMR at any time*	57/68 (83)
*NGS MRD negative after cycle 1*	17/36 (47)
*NGS MRD negative at any time*	55/57 (96)

Abbreviations: WBC, white blood cell count; CR, complete remission; CRi, complete remission with incomplete hematologic recovery; MRD, measurable residual disease; CMR, complete molecular response; NGS, next-generation sequencing

^1^ Risk factors include: hypertension, hyperlipidemia, diabetes and/or coronary artery disease

^2^ Includes patients with an *IKZF1* deletion or *IKZF1* frameshift mutation in combination with a *CDKN2A/B* and/or *PAX5* deletion

^3^ Excludes patients who were in morphological remission at the time of enrollment

^4^ Excludes patients who were MRD-negative at the time of enrollment

### Response, disposition, and survival outcomes

Response rates for the study cohort are shown in Table 1. Among 53 patients with active disease at study enrollment, 52 patients (98%) achieved complete remission (CR) or CR with incomplete hematologic recovery (CRi). One patient experienced early death due to intracranial hemorrhage. The CMR rate after cycle 1 was 59%, and the overall CMR rate was 83%. The rate of NGS MRD negativity after cycle 1 was 47%, and the overall NGS MRD negativity rate was 96%. Among 8 patients who did not achieve CMR and were also assessed by NGS MRD, all were NGS MRD negative.

Patient disposition is shown in Supplemental Fig. 1. Among the 76 patients, 3 patients (4%) died in CR, 1 patient (1%) died in cycle 1 prior to response assessment, 10 patients (13%) relapsed, 2 patients (3%) underwent alloSCT in first remission (both due to lack of achievement of CMR), and 58 patients (76%) are in ongoing continuous remission without alloSCT. Three of these patients were taken off protocol to receive additional therapy for MRD-positive disease (inotuzumab ozogamicin, *n* = 1; CD19 CAR T-cells, *n* = 2).

With a median follow-up of 29 months (range, 5–75 months), the median EFS and OS has not been reached. Seven patients have died: 1 early death during induction, 3 deaths in CR, and 3 due to ALL relapse. The estimated 2-year and 3-year CIR are 15% and 17%, respectively, the 2-year and 3-year EFS rates are 80% and 78%, respectively, and the 2-year and 3-year OS rates are 93% and 88%, respectively (Supplemental Fig. 2). In a *post hoc* analysis of “MRD-based EFS” considering NGS MRD persistence or recurrence as an event (3 additional patients), the 2-year and 3-year EFS rates were 78% and 76%, respectively (Supplemental Fig. 3).

### Molecular characteristics of the study population

**Fig. 1:**
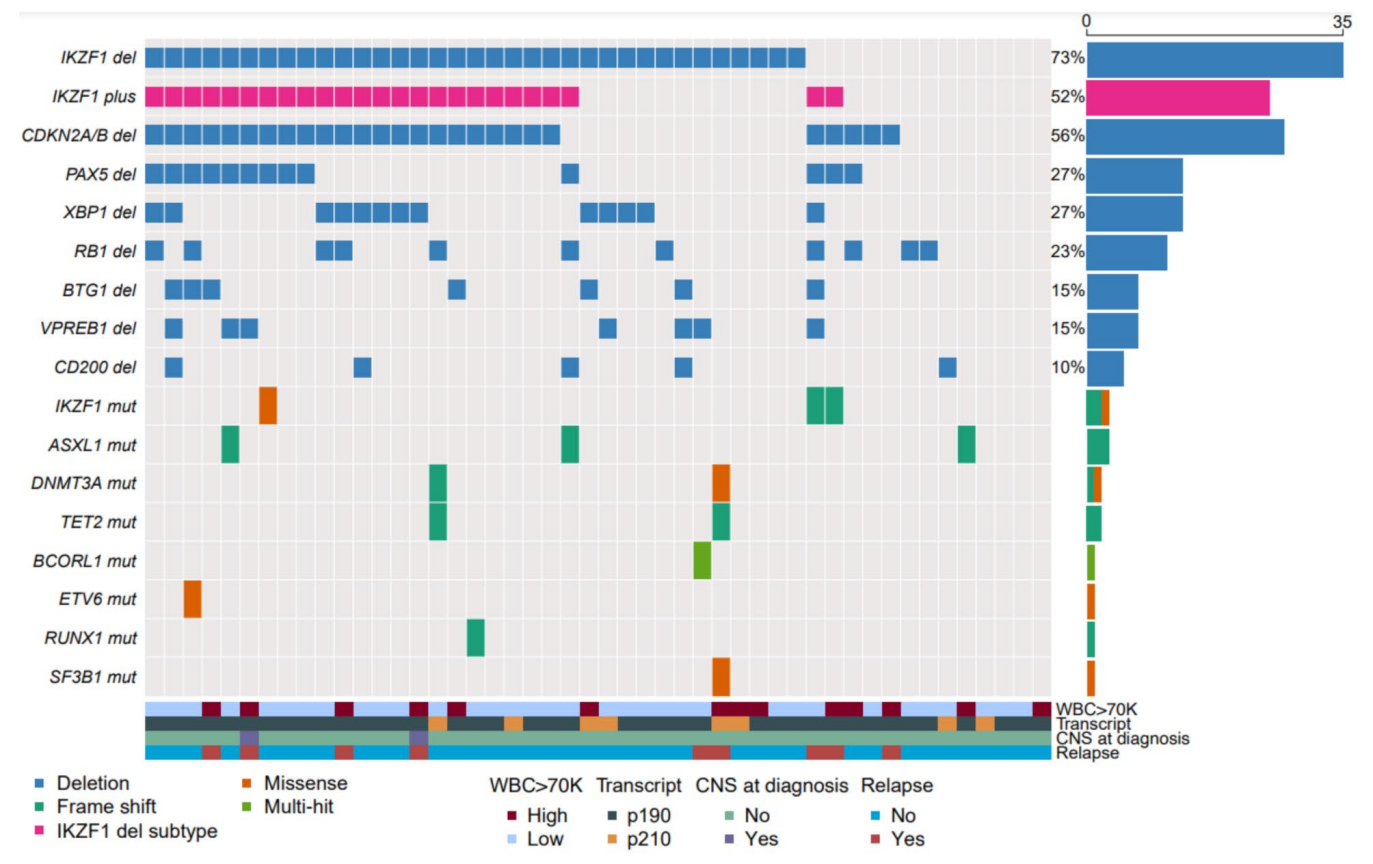
Oncoplot of patients with complete molecular profiling (*n* = 48)

Overall, 57 patients (75%) underwent baseline targeted sequencing, and 48 patients (63%) underwent SNP microarray. There were no significant differences in clinical characteristics between patients who underwent SNP array versus those who did not, although patients with SNP data tended to be older (*P* = 0.10) and have higher WBC at diagnosis (*P* = 0.13) (Supplemental Table 2). The incidence of individual genomic alterations in the study population is shown in Table 1. Among patients who underwent targeted sequencing, the most common mutations were *IKZF1* in 5 patients (9%), *ASXL1*, *DNMT3A*, and *TET2* in 3 patients (5%) each, and *BCORL1*, *ETV6*, *RUNX1*, and *SF3B1* in 1 patient (2%) each. Among the 5 IKZF1 mutations, 2 were frameshift and 3 were missense. Among patients who underwent SNP microarray, the most common alterations were an *IKZF1* deletion (73%), *CDK2NA/B* deletion (56%), and *PAX5* and *XBP1* deletions (27% each). Fourteen of the *IKZF1* deletions were dominant negative (40% of total *IKZF1* deletions) [9]. Seven patients (15%) had a *VPREB1* deletion. Twenty-five patients (52%) were classified as having the *IKZF1*^plus^ genotype (defined as an *IKZF1* deletion or frameshift mutation in combination with deletion of *CDKN2A/B* and/or *PAX5*). An Oncoplot of the 48 patients with complete molecular information, WBC count at diagnosis, transcript type, CNS involvement at diagnosis, and relapse status is shown in Fig. 1.

### Relapse characteristics

The clinical and molecular characteristics of the 10 patients who relapsed are shown in Table 2. One patient who relapsed did not have an available baseline sample for SNP array. The median age at relapse was 46 years (range, 18–70 years), and the median WBC at diagnosis of the relapsed patients was 124.1 × 10^9^/L (range, 12.9-322.1 × 10^9^/L). The median time to relapse was 18 months (range, 8–24 months). Four relapses occurred in the bone marrow, with a median time to relapse of 14 months (range, 8–24 months). At relapse, one patient had an *ABL1* kinase domain (KD) E255V mutation, one had a T3151 mutation (in a patient who was on dasatinib at the time of relapse due to the development of worsening coronary atherosclerosis), and two did not have an *ABL1* KD mutation detected. One patient with bone marrow relapse had concomitant leukemic involvement of the vitreous fluid. Five relapses occurred in the CNS only, with a median time to relapse of 21 months (range, 9–23 months). One patient experienced a non-CNS extramedullary relapse. This patient had an *IGH::CRLF2* fusion at diagnosis and relapsed in the peritoneum and lymph nodes with Ph-negative disease. All tested patients retained high levels of CD19 expression at relapse (median 99.9% [range, 82.3-100%]).

**Table 2:** – Clinical and molecular characteristics of relapsed patients

Patient	Age	WBC at diagnosis (x 10^9^/L)	Transcript type	Mutations (targeted sequencing)	Gene deletions (SNP array)	NGS MRD response after C1	PCR MRD response after C1	Duration of CR1 (months)	Type of relapse
#1	57	2.0	p190	*IKZF1*	*CDKN2A/B*, *PAX5*, *VPREB1*,* BTG1*,* RB1*,* XBP1*	Negative	CMR	8.6	Peritoneum and lymph nodes (Ph-negative)
#2	60	322.1	p190	*IKZF1*	*CDKN2A/B*,* PAX5*	Not done	CMR	24.5	Bone marrow
#3	44	152.6	p190	None	*CDKN2A/B*	Positive (1/million)	CMR	7.6	Bone marrow
#4	18	4.5	p190	None	Not done	Positive (below LOD)	CMR	11.3	Bone marrow
#5	48	95.5	p190	None	*IKZF1*,* CDKN2A/B*,* PAX5*,* BTG1*	Not done	Not done	17.0	Bone marrow + vitreous fluid
#6	28	270.5	p190	Not done	*IKZF1*,* CDKN2A/B*,* PAX5*,* VPREB1*	Not done	CMR	22.0	CNS
#7	43	12.9	p190	*BCORL1*	*IKZF1*,* VPREB1*	Negative	CMR	19.8	CNS
#8	49	84.9	p190	None	*IKZF1*,* CDKN2A/B*,* RB1*,* XBP1*	Not done	CMR	23.2	CNS
#9	44	236.7	p190	None	*IKZF1*,* CDKN2A/B*,* XBP1*	Positive (57/million)	CMR	8.5	CNS
#10	70	181.2	p210	*DNMT3A*,* SF3B1*,* TET2*	*IKZF1*	Positive (below LOD)	CMR	20.7	CNS

Abbreviations: WBC, white blood cell; SNP, single nucleotide polymorphism; NGS, next-generation sequencing; MRD, measurable residual disease; C1, cycle 1; LOD, level of detection; PCR, polymerase chain reaction; CMR, complete molecular response; CR1, first remission; Ph, Philadelphia chromosome; CNS, central nervous system

All relapsed patients received salvage therapy. The salvage therapies administered and responses are shown in Supplemental Table 3. Nine patients (90%) achieved complete remission after first salvage therapy, and one patient died during reinduction due to complications from CNS relapse. Five of the responding patients received subsequent CD19 chimeric antigen receptor T-cell as consolidation, none of whom has subsequently relapsed. No patient underwent alloSCT following relapse.

### Predictors of relapse

**Fig. 2:**
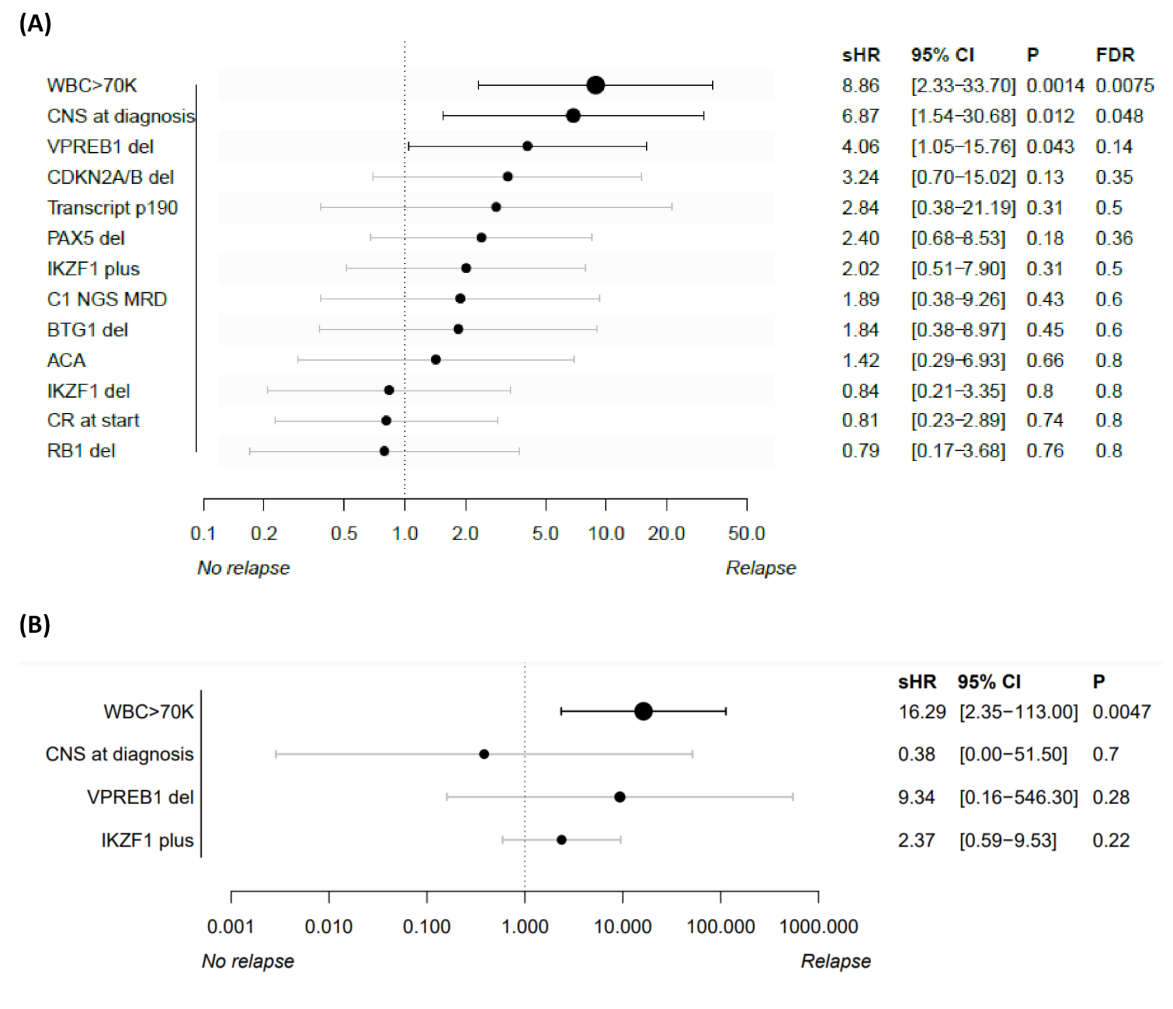
Predictors for relapse with blinatumomab and ponatinib in newly diagnosed Ph + ALL. (**A**) Forest plot for univariate analysis and (**B**) Forest plot for multivariate analysis

On univariate analysis, factors associated with an increased risk of relapse on univariate analysis included: WBC ≥ 70 × 10^9^/L at diagnosis (sHR 8.86 [95% CI 2.33–33.70]; *P* = 0.001), presence of CNS involvement at diagnosis (sHR 6.87 [95% CI 1.54–30.68]; *P* = 0.01), and *VPREB1* deletion (sHR 4.06 [95% CI 1.05–15.76]; *P* = 0.04) (Fig. 2A**)**. Among 69 patients who were evaluable for complete molecular response (CMR) after cycle 1, there were 9 relapses; paradoxically, all these relapses occurred in patients who achieved early CMR. The 3-year CIR for patients with *IKZF1*^plus^ was 29% and for patients without the *IKZF1*^plus^ genotype was 14% (sHR 2.02 [95% CI 0.51–7.9]; *P* = 0.31) (Supplemental Fig. 4). Similarly, neither transcript type (p190 vs. p210: sHR 2.84 [95% CI 0.38–21.19; *P* = 0.31) nor NGS MRD positivity after cycle 1 (sHR 1.89 [95% CI 0.38–9.26; *P* = 0.43) significantly impacted the risk of relapse (Supplemental Figs. 5–6).

**Fig. 3:**
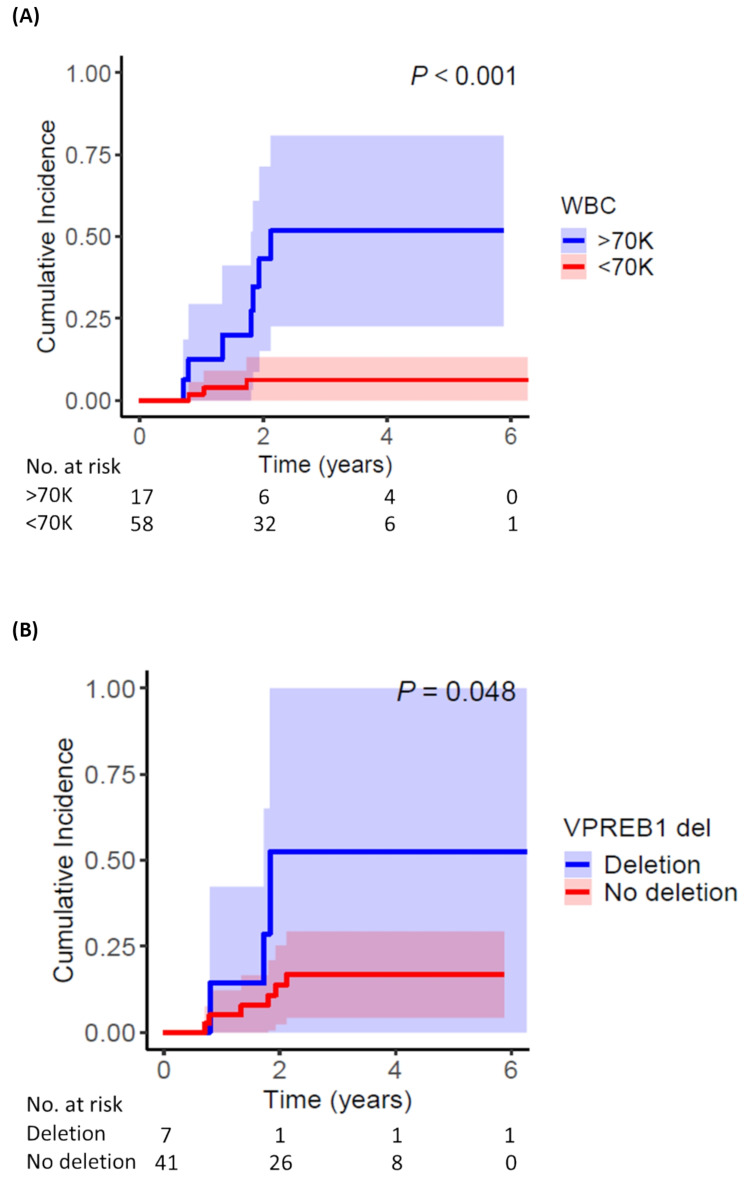
Cumulative incidence of relapse by subgroup. (**A**) WBC count at diagnosis and (**B**) *VPREB1* deletion status. The 95% confidence interval is represented by the shaded areas. The unadjusted *P* value is shown

Overall, 17 patients (22% of the entire cohort) had WBC ≥ 70 × 10^9^/L at the time of diagnosis. The CIR for patients with WBC ≥ 70 versus < 70 × 10^9^/L was 53% and 6%, respectively **(**Fig. 3A**).** The CIR for patients with *VPREB1* deletion versus no deletion was 52% and 17%, respectively **(**Fig. 3B**).** Notably, only 3 of the 58 patients with WBC < 70 × 10^9^/L relapsed; 2 of these patients had a *VPREB1* deletion and the other patient did not have a sample available for testing (this patient was also non-compliant and relapsed while off all therapy for 11 months). Thus, among 9 patients with full clinical and molecular data who relapsed, all relapses could be explained by either high WBC at presentation or by the presence of a *VPREB1* deletion. Neither Pearson nor Spearman correlations indicated a statistically significant association among WBC ≥ 70 × 10⁹/L, *IKZF1*^*plus*^ genotype, and *VPREB1* deletion. On MVA, only WBC ≥ 70 × 10^9^/L at diagnosis was significantly associated with relapse risk (sHR 16.29 [95% CI 2.35–113.00]; *P* = 0.005 **(**Fig. 2B**).**

## Discussion

Various prognostic factors have been previously described in Ph + ALL, including high-risk additional chromosomal abnormalities, *IKZF1*^plus^ genotype, and MRD response [10, 11, 12]. However, most studies were performed in the context of regimens combining intensive chemotherapy with a first- or second-generation BCR::ABL1 TKI. In this analysis of patients treated uniformly with a chemotherapy-free regimen of blinatumomab and ponatinib, only WBC ≥ 70 × 10^9^/L was independently associated with an increased risk of relapse, superseding the prognostic value of early MRD clearance and baseline genomic alterations such as *IKZF1*^plus^ genotype.

The strong prognostic impact of high WBC in this cohort (CIR of 53%) may be at least partially explained by the chemotherapy-free nature of the regimen, which omits high-dose systemic methotrexate and cytarabine and therefore could predispose to a higher relative risk of CNS relapses, which accounted for half of the relapses in our study [13]. A similar proportion and rate of CNS relapses was also observed in the D-ALBA study [2]. In contrast, in a study of intensive chemotherapy with hyper-CVAD plus ponatinib in 86 patients with newly diagnosed Ph + ALL, 15 relapses were observed, none of which occurred in the CNS [14]. Other recent studies have suggested that omission of high-dose cytarabine in consolidation may increase the risk of relapse in Ph + ALL, which is consistent with our findings [15]. Additional strategies to prevent CNS relapses are therefore needed for patients with Ph + ALL receiving frontline chemotherapy-free regimens. To attempt to mitigate this risk of CNS relapse, we have amended the blinatumomab and ponatinib study to recommend 2 cycles of high-dose methotrexate and cytarabine to patients with WBC ≥ 70 × 10^9^/L at presentation. All patients now also receive 15 doses of intrathecal chemotherapy, which was increased from 12 in the initial version of the protocol. A similar strategy of more intensive intrathecal prophylaxis has also been adopted by other groups evaluating chemotherapy-free blinatumomab and TKI combinations in newly diagnosed Ph + ALL [16].

Several previous analyses have identified the *IKZF1*^plus^ genotype as a high-risk feature in Ph + ALL, including in studies of hyper-CVAD plus ponatinib and with blinatumomab plus dasatinib [2, 17]. However, in our analysis, *IKZF1*^plus^ genotype was not significantly associated with relapse risk. A limitation of our study is the relatively small number of relapses (*n* = 10). Given that our study was underpowered to detect predictors of relapse risk with small to moderate effect sizes, it is possible that an association could be observed with more patients and longer follow-up. If the lack of prognostic impact of *IKZF1*^plus^ is observed in other studies evaluating blinatumomab and ponatinib (e.g. the ongoing GIMEMA ALL2820 study) [16], this would suggest that this combination can overcome the poor-risk biology of this ALL subtype. However, at present, our findings are only hypothesis-generating.

The lack of association of MRD clearance and clinical outcomes is unexpected, given the strong association of MRD with relapse risk in many other ALL studies, including in Ph + ALL [2, 12, 18, 19]. The rates of both CMR and NGS MRD negativity were very high in our study (83% and 96%, respectively), which makes it challenging to compare outcomes according to best MRD response. Furthermore, recent data suggest the PCR for *BCR::ABL1* may be a suboptimal method of MRD assessment in many patients with Ph + ALL [19, 20]. Interestingly, early MRD dynamics (e.g. MRD response after cycle 1) also did not predict relapse risk. Rather, we found that only baseline clinical characteristics—particularly high WBC at presentation—were independently associated with risk of relapse.

We observed a high risk of relapse in patients with a *VPREB1* deletion (CIR of 52%), although this association was not statistically significant on MVA. *VPREB1* is involved in normal B-cell development and encodes a surrogate light chain in the pre-B-cell receptor complex [21]. *VPREB1* deletion has been associated with worse outcomes in ALL, although most of these analyses did not did specifically evaluate its role in Ph + ALL [22, 23]. Given the small number of patients with *VPREB1* deletion and the lack of association of *VPREB1* on multivariate analysis, these results should be interpreted with caution. Future studies are needed to clarify the possible prognostic impact of *VPREB1* in Ph + ALL with different treatment approaches, including both chemotherapy-containing and chemotherapy-free regimens.

## Conclusions

In conclusion, we observed that patients with Ph + ALL who have WBC ≥ 70 × 10^9^/L at diagnosis have a very high relative risk of relapse with frontline blinatumomab and ponatinib. Contrary to other studies, neither *IKZF1*^plus^ genotype nor MRD dynamics impacted relapse risk. High WBC at presentation appears to be the strongest prognostic factor for patients with Ph + ALL receiving frontline chemotherapy-free regimens, although this finding—as well as the lack of prognostic significance with *IKZF1*^plus^ genotype and MRD response—will need to be validated in a larger independent cohort. An increase in the number of doses of intrathecal chemotherapy prophylaxis and incorporation of high-dose methotrexate and cytarabine are being evaluated prospectively in an attempt to reduce the risk of relapse in this subgroup.

## Electronic supplementary material

Below is the link to the electronic supplementary material.

Supplementary Material 1

## Data Availability

The study data is not publicly available to respect participant confidentiality. Requests for sharing of deidentified data should be directed to the corresponding author.

## Abbreviations

ALL: Acute lymphoblastic leukemia
TKI: Tyrosine kinase inhibitor
alloSCT: Allogeneic stem cell transplant
DFS: Disease-free survival
OS: Overall survival
CNS: Central nervous system
SNP: Single-nucleotide polymorphism
CMR: Complete molecular response
FDR: False discovery rate
CIR: Cumulative incidence of relapse
EFS: Event-free survival
MVA: Multivariate analysis
sHR: Subdistribution hazard ratios
CR: Complete remission
CRi: CR with incomplete hematologic recovery
KD: Kinase domain

## References

[1] Foà R, Chiaretti S. Philadelphia Chromosome-Positive acute lymphoblastic leukemia. N Engl J Med. 2022;386:2399–411. 35731654 10.1056/NEJMra2113347

[2] Foà R, Bassan R, Elia L, et al. Long-Term results of the Dasatinib-Blinatumomab protocol for adult Philadelphia-Positive ALL. J Clin Oncol. 2024;42:881–5. 38127722 10.1200/JCO.23.01075 PMC10927329

[3] Kantarjian H, Short NJ, Haddad FG et al. Results of the simultaneous combination of Ponatinib and blinatumomab in Philadelphia Chromosome-Positive ALL. J Clin Oncol 0:JCO.24.00272. 10.1200/JCO.24.00272 PMC12360455 39028925

[4] Jabbour E, Short NJ, Jain N, et al. Ponatinib and blinatumomab for Philadelphia chromosome-positive acute lymphoblastic leukaemia: a US, single-centre, single-arm, phase 2 trial. Lancet Haematol. 2023;10:e24–34. 36402146 10.1016/S2352-3026(22)00319-2

[5] Short NJ, Kantarjian HM, Loghavi S, et al. Treatment with a 5-day versus a 10-day schedule of decitabine in older patients with newly diagnosed acute myeloid leukaemia: a randomised phase 2 trial. Lancet Haematol. 2019;6:e29–37. 30545576 10.1016/S2352-3026(18)30182-0 PMC6563344

[6] Luthra R, Patel KP, Reddy NG, et al. Next-generation sequencing-based multigene mutational screening for acute myeloid leukemia using miseq: applicability for diagnostics and disease monitoring. Haematologica. 2014;99:465–73. 24142997 10.3324/haematol.2013.093765 PMC3943309

[7] Mermel CH, Schumacher SE, Hill B, et al. GISTIC2.0 facilitates sensitive and confident localization of the targets of focal somatic copy-number alteration in human cancers. Genome Biol. 2011;12:R41. 21527027 10.1186/gb-2011-12-4-r41 PMC3218867

[8] Daenekas B, Pérez E, Boniolo F et al. Conumee 2.0: enhanced copy-number variation analysis from DNA methylation arrays for humans and mice. Bioinformatics 40, 2024. 10.1093/bioinformatics/btae029 PMC10868300 38244574

[9] Mullighan CG, Su X, Zhang J, et al. Deletion of IKZF1 and prognosis in acute lymphoblastic leukemia. N Engl J Med. 2009;360:470–80. 19129520 10.1056/NEJMoa0808253 PMC2674612

[10] Short NJ, Kantarjian HM, Sasaki K, et al. Poor outcomes associated with + der(22)t(9;22) and– 9/9p in patients with Philadelphia chromosome-positive acute lymphoblastic leukemia receiving chemotherapy plus a tyrosine kinase inhibitor. Am J Hematol. 2017;92:238–43. 28006851 10.1002/ajh.24625 PMC5495018

[11] Chiaretti S, Ansuinelli M, Vitale A, et al. A multicenter total therapy strategy for de Novo adult Philadelphia chromosome positive acute lymphoblastic leukemia patients: final results of the GIMEMA LAL1509 protocol. Haematologica. 2021;106:1828–38. 33538150 10.3324/haematol.2020.260935 PMC8252956

[12] Short NJ, Jabbour E, Sasaki K, et al. Impact of complete molecular response on survival in patients with Philadelphia chromosome-positive acute lymphoblastic leukemia. Blood. 2016;128:504–7. 27235138 10.1182/blood-2016-03-707562 PMC4965905

[13] Paul S, Short NJ. Central nervous system involvement in adults with acute leukemia: diagnosis, prevention, and management. Curr Oncol Rep. 2022;24:427–36. 35141858 10.1007/s11912-022-01220-4

[14] Kantarjian H, Short NJ, Jain N, et al. Frontline combination of Ponatinib and hyper-CVAD in Philadelphia chromosome-positive acute lymphoblastic leukemia: 80-months follow-up results. Am J Hematol. 2023;98:493–501. 36600670 10.1002/ajh.26816 PMC12777642

[15] Chalandon Y, Rousselot P, Chevret S, et al. Nilotinib with or without cytarabine for Philadelphia-positive acute lymphoblastic leukemia. Blood. 2024;143:2363–72. 38452207 10.1182/blood.2023023502

[16] Chiaretti S, Leoncin M, Elia L, et al. Efficacy and toxicity of frontline Ponatinib plus blinatumomab for adult Ph + ALL patients of all ages. Intermediate analysis of the Gimema ALL2820. Blood. 2024;144:835–835.

[17] Sasaki Y, Kantarjian HM, Short NJ, et al. Genetic correlates in patients with Philadelphia chromosome-positive acute lymphoblastic leukemia treated with Hyper-CVAD plus dasatinib or Ponatinib. Leukemia. 2022;36:1253–60. 35132195 10.1038/s41375-021-01496-8 PMC12001897

[18] Berry DA, Zhou S, Higley H, et al. Association of minimal residual disease with clinical outcome in pediatric and adult acute lymphoblastic leukemia: A Meta-analysis. JAMA Oncol. 2017;3:e170580–170580. 28494052 10.1001/jamaoncol.2017.0580 PMC5824235

[19] Short NJ, Jabbour E, Macaron W, et al. Ultrasensitive NGS MRD assessment in Ph + ALL: prognostic impact and correlation with RT-PCR for BCR::ABL1. Am J Hematol. 2023;98:1196–203. 37183966 10.1002/ajh.26949

[20] Zuna J, Hovorkova L, Krotka J, et al. Minimal residual disease in BCR::ABL1-positive acute lymphoblastic leukemia: different significance in typical ALL and in CML-like disease. Leukemia. 2022;36:2793–801. 35933523 10.1038/s41375-022-01668-0

[21] Reth M, Nielsen P. Signaling circuits in early B-cell development. Adv Immunol. 2014;122:129–75. 24507157 10.1016/B978-0-12-800267-4.00004-3

[22] Mangum DS, Downie J, Mason CC, et al. VPREB1 deletions occur independent of lambda light chain rearrangement in childhood acute lymphoblastic leukemia. Leukemia. 2014;28:216–20. 23881307 10.1038/leu.2013.223 PMC4043450

[23] Miles RR, Downie J, Jahromi MS, et al. VPREB1 deletions occur independent of Lambda-Light chain rearrangement and predict worse outcome in pediatric acute lymphoblastic leukemia (ALL). Blood. 2010;116:273–273.

