# Supplementary material for: Molecular characterization and predictors of relapse in patients with Ph + ALL after frontline ponatinib and blinatumomab
Source: J Hematol Oncol. 2025 May 14;18:55. doi: 10.1186/s13045-025-01709-y (PMC12079890; doi:10.1186/s13045-025-01709-y)
Supplement: Supplementary file 1 — Supplementary Material 1 [file 13045_2025_1709_MOESM1_ESM.docx]

**Supplemental Table 1 – Mutations included in the 81-gene targeted sequencing panel**

| *ANKRD26* | *CBLB* | *EED* | *GFI1* | *JAK1* | *NF1* | *PTEN* | *SH2B3* | *SUZ12* |
| --- | --- | --- | --- | --- | --- | --- | --- | --- |
| *ASXL1* | *CBLC* | *ELANE* | *GNAS* | *JAK2* | *NOTCH1* | *PTPN11* | *SMC1A* | *TERC* |
| *ASXL2* | *CEBPA* | *ETNK1* | *HNRNPK* | *JAK3* | *NPM1* | *RAD21* | *SMC3* | *TERT* |
| *BCOR* | *CREBBP* | *ETV6* | *HRAS* | *KDM6A* | *NRAS* | *RARA* | *SRSF2* | *TET2* |
| *BCORL1* | *CRLF2* | *EZH2* | *IDH1* | *KIT* | *PAX5* | *RUNX1* | *STAG1* | *TP53* |
| *BRAF* | *CSF3R* | *FBXW7* | *IDH2* | *KMT2A* | *PHF6* | *SETBP1* | *STAG2* | *U2AF1* |
| *BRINP3* | *CUX1* | *FLT3* | *IKZF1* | *KRAS* | *PIGA* | *SF1* | *STAT3* | *U2AF2* |
| *CALR* | *DDX41* | *GATA1* | *IL2RG* | *MAP2K1* | *PML* | *SF3A1* | *STAT5A* | *WT1* |
| *CBL* | *DNMT3A* | *GATA2* | *IL7R* | *MPL* | *PRPF40B* | *SF3B1* | *STAT5B* | *ZRSR2* |

**Supplemental Table 2 – Baseline characteristics stratified by single-nucleotide polymorphism (SNP) microarray testing**

| **Baseline characteristics** | **n/N (%) / median [range]** | | |
| --- | --- | --- | --- |
|  | SNP data available (n=48) | No SNP data (n=28) | p-value |
| Age (years)  *Age ≥ 60 years* | 56 [20-83]  21 (44) | 45 [18-79]  7 (25) | p=0.10  p=0.10 |
| Baseline cardiovascular risk factor^1^ | 29 (60) | 11 (39) | p=0.08 |
| WBC at diagnosis (x10^9^/L)  *WBC at diagnosis ≥ 70 x10^9^/L* | 19.3 [0.7-322.1]  14 (29) | 8.2 [0.6-254.7]  3 (11) | p=0.13  p=0.06 |
| Central nervous system involvement at diagnosis | 2 (4) | 1 (4) | p=0.90 |
| CD19 expression (%) | 99.8 [74.9-100] | 99.5 [89.6-99.9] | p=0.68 |
| *BCR::ABL1* transcript  *p190*  *p210* | 40 (83)  8 (17) | 20/27 (74)  7/27 (26) | p=0.34 |
| Gene mutations  *IKZF1*  *ASXL1*  *DNMT3A*  *TET2*  *BCORL1*  *ETV6*  *RUNX1*  *SF3B1* | 3/44 (7)  3/44 (7)  2/44 (5)  2/44 (5)  1/44 (2)  1/44 (2)  1/44 (2)  1/44 (2) | 2/13 (15)  0  1/13 (8)  1/13 (8)  0  0  0  0 | p=0.34  --  p=0.66  p=0.66  --  --  --  -- |

Abbreviations: SNP, single-nucleotide polymorphism; WBC, white blood cell count

**^1^** Risk factors include: hypertension, hyperlipidemia, diabetes and/or coronary artery disease

**Supplemental Table 3 – Salvage therapies and outcomes in relapsed patients**

| **Patient** | **Type of relapse** | **First salvage regimen** | **Response** | **Consolidation** | **Second relapse** | **Vital status** |
| --- | --- | --- | --- | --- | --- | --- |
| #1 | Peritoneum and lymph nodes (Ph-negative) | Mini-hyper-CVD + INO + blina + dasatinib | CR | Brexu-cel | No | Dead |
| #2 | Bone marrow | Min-hyper-CVD + INO + blina + nilotinib | CR | Brexu-cel | No | Alive |
| #3 | Bone marrow | Mini-hyper-CVD + INO + blina + ponatinib | CR | N/A | Yes | Dead |
| #4 | Bone marrow | Blina + asciminib | CR | N/A | No | Alive |
| #5 | Bone marrow + vitreous fluid | FA + INO + blina + dasatinib | CR | N/A | No | Alive |
| #6 | CNS | HD-MTX/Ara-C + dasatinib + IT 🡪 XRT | CR | Brexu-cel | No | Alive |
| #7 | CNS | HD-MTX/Ara-C + dasatinib + IT | Died | N/A | N/A | Dead |
| #8 | CNS | XRT 🡪 HD-MTX/Ara-C + dasatinib + IT | CR | Brexu-cel | No | Alive |
| #9 | CNS | HD-MTX/Ara-C + INO + ponatinib + IT 🡪 XRT | CR | Brexu-cel | No | Alive |
| #10 | CNS | Ponatinib + IT | CR | N/A | No | Alive |

Abbreviations: Ph, Philadelphia chromosome; CNS, central nervous system; mini-hyper-CVD, mini-hyperfractionated cyclophosphamide, vincristine and dexamethasone alternating with mini-methotrexate and cytarabine; INO, inotuzumab ozogamicin; blina, blinatumomab; FA, fludarabine and cytarabine; IT, intrathecal chemotherapy; XRT, irradiation; CR, complete remission; brexu-cel, brexucabtagene autoleucel; N/A, not applicable

**Supplemental Figure 1 – Patient disposition**


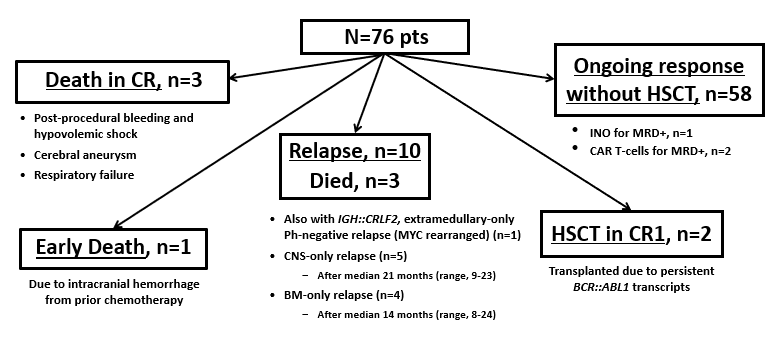


**Supplemental Figure 2 – Outcomes of the study cohort.** (A) Cumulative incidence of relapse, (B) Event-free survival, and (C) Overall survival.

**(A)**

**
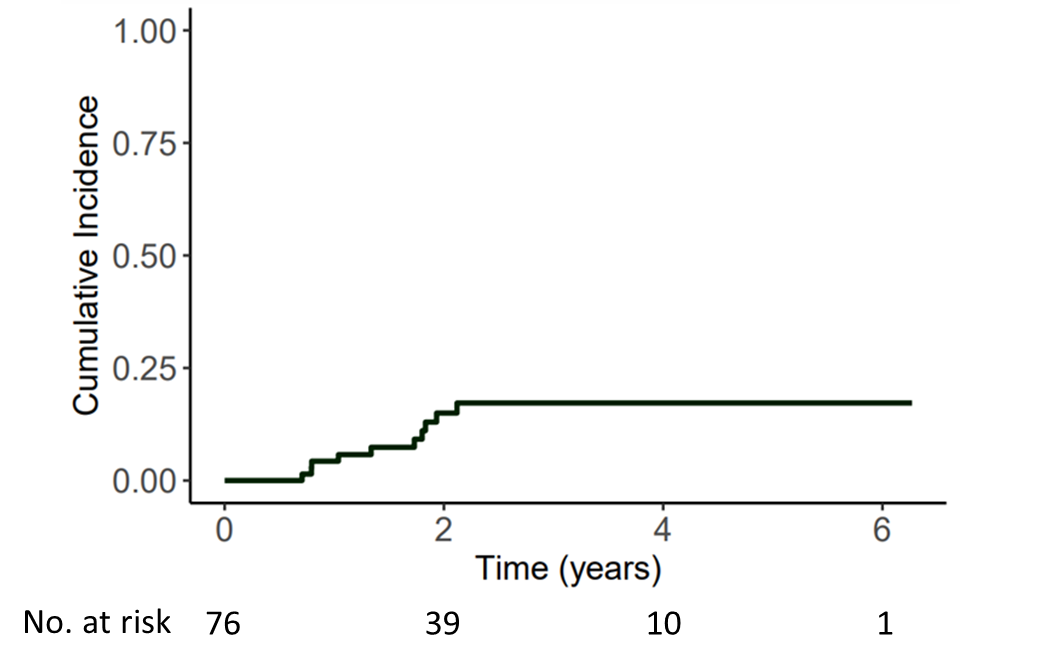
**

**(B)**

**
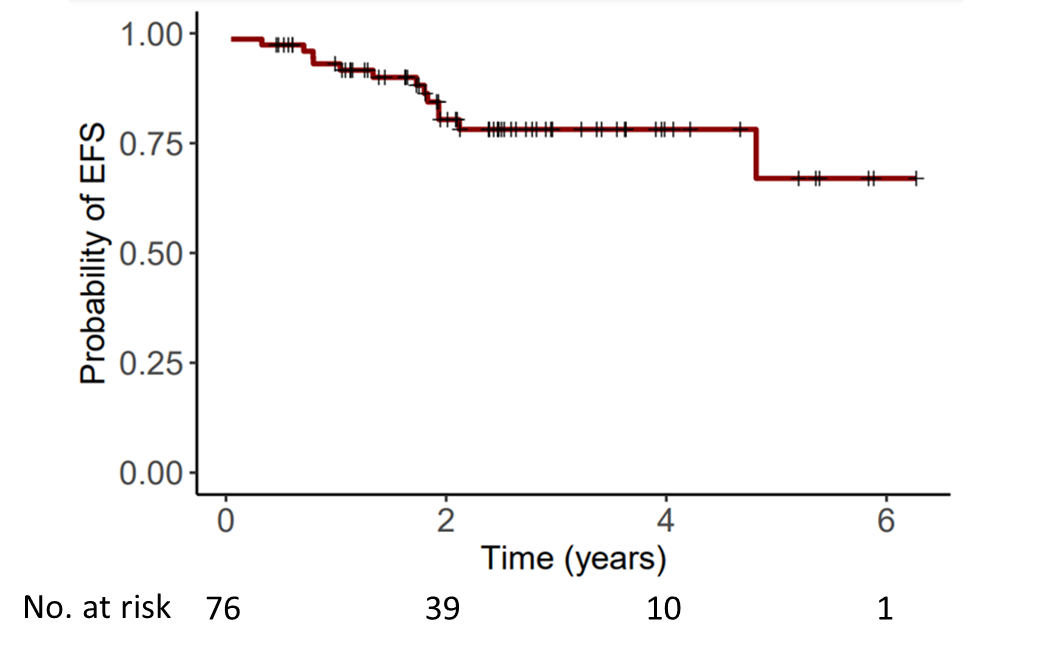
**

**(C)**

**
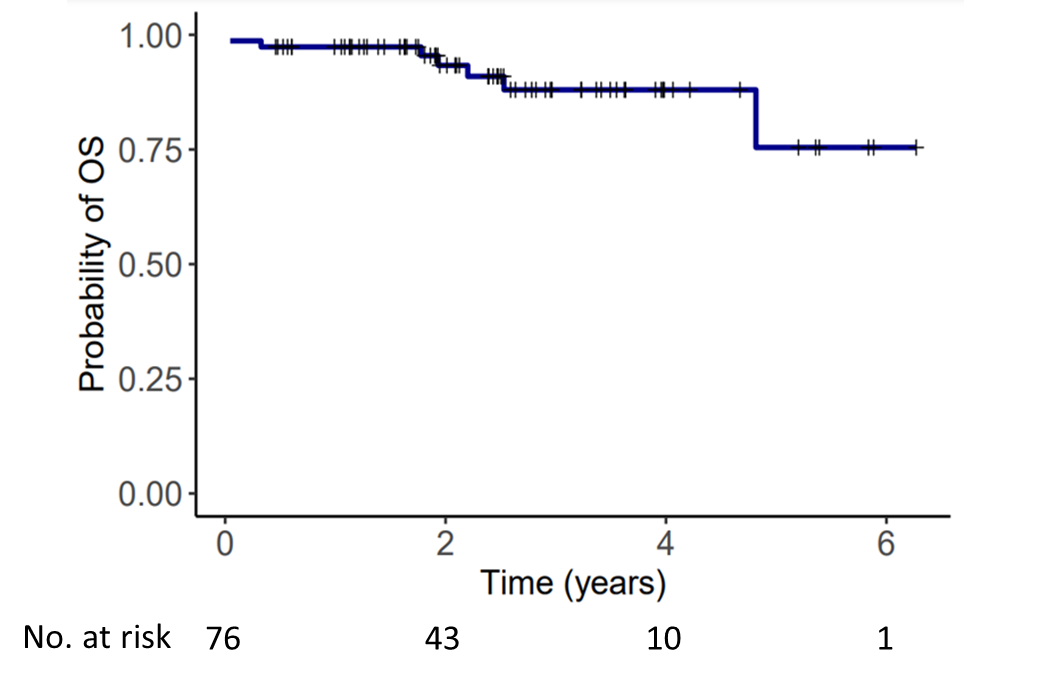
**

**Supplemental figure 3 –** **Event-free survival of the study cohort, considering NGS MRD persistence or recurrence as an event.**


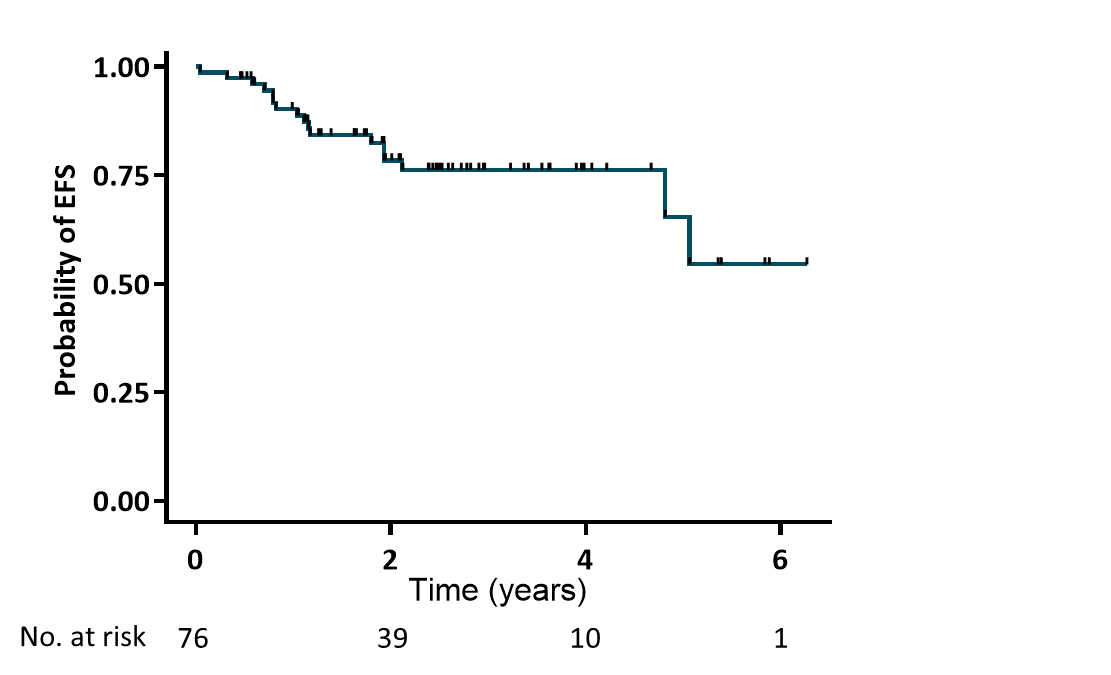


**Supplemental Figure 4 – Cumulative incidence of relapse by stratified by *IKZF1*^plus^ deletion status.** The 95% confidence interval is represented by the shaded areas. The unadjusted P value is shown.

**
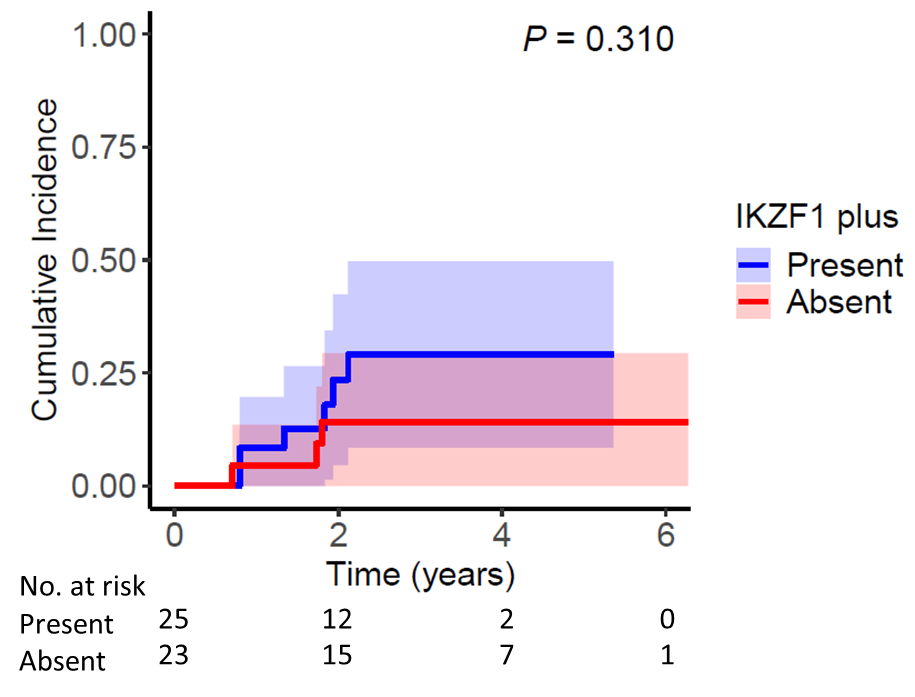
**

**Supplemental Figure 5 – Cumulative incidence of relapse, stratified by BCR::ABL1 transcript type.** The 95% confidence interval is represented by the shaded areas. The unadjusted P value is shown.

**
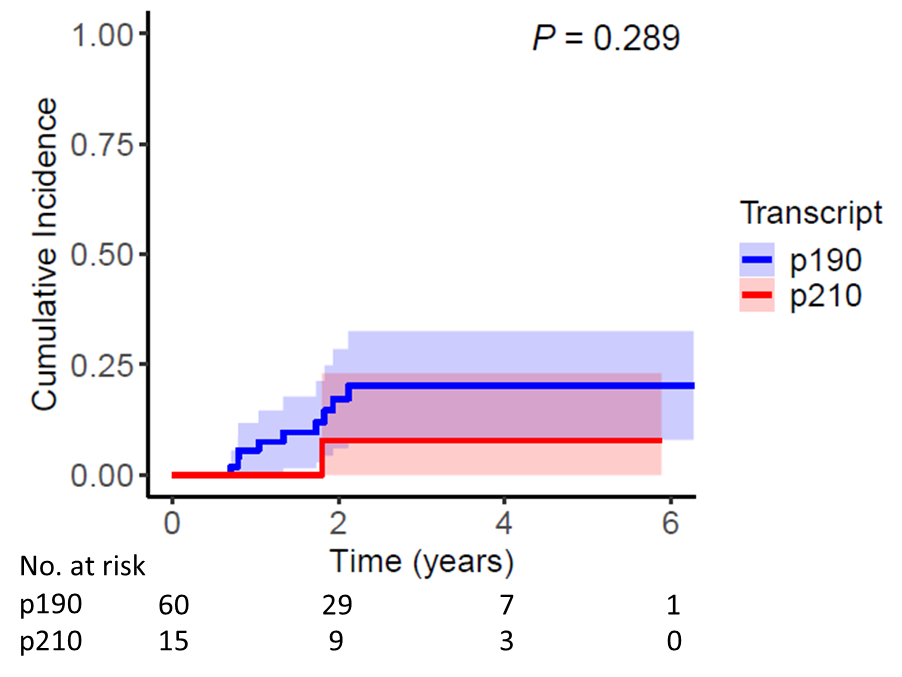
**

**Supplemental Figure 6 – Cumulative incidence of relapse by NGS MRD negativity after cycle 1.** The 95% confidence interval is represented by the shaded areas. The unadjusted P value is shown.

**
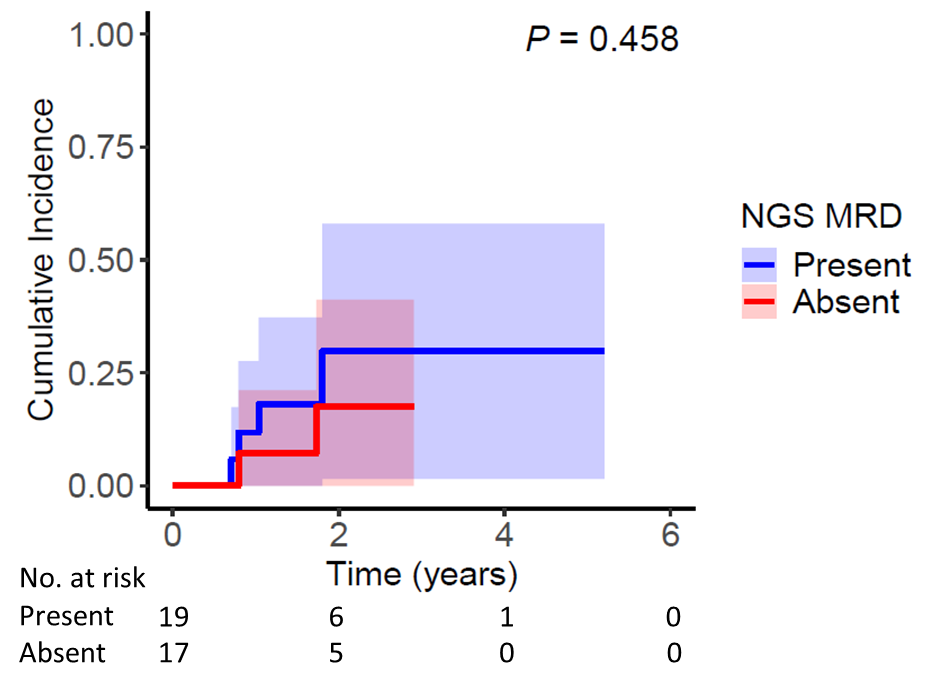
**

**Supplemental Material – Clinical trial protocol**

**Phase II Study of the Combination of Blinatumomab and Ponatinib in Patients with Philadelphia Chromosome (Ph)-Positive and/or BCR-ABL Positive Acute Lymphoblastic Leukemia (ALL)**

**MDACC Protocol #:** 2016-0792

**Sponsor:** MD Anderson

**Principal Investigator:** Elias Jabbour, MD Co-Principal Investigator:

Biostatistical Collaborators: Xuelin Huang

**Address:** 1400 Holcombe Blvd, Unit 428

Houston, TX 77030

**Phone:** 713-792-4764

**Fax:** 713-563-7746

**Email:** [ejabbour@mdanderson.org](mailto:ejabbour@mdanderson.org)

**Protocol Version** **27**

**Version Date 12/02/2024**

**IND Sponsor The University of Texas MD Anderson Cancer Center IND #** **131253**

**ClinicalTrials.gov #:**

**TABLE OF CONTENTS**

1. **INTRODUCTION** **3**
2. [**STUDY OBJECTIVES** **12**](#_TOC_250008)
3. [**SELECTION OF PATIENTS** **12**](#_TOC_250007)
   1. [**Inclusion Criteria** **12**](#_TOC_250006)
   2. [**Exclusion Criteria** **14**](#_TOC_250005)
4. [**TREATMENT OF SUBJECTS** **15**](#_TOC_250004)
5. [**CONCOMITTANT MEDICATIONS** **29**](#_TOC_250003)
6. **STUDY PROCEDURES** **30**
7. [**EFFICACY AND SAFETY ASSESSMENTS** **34**](#_TOC_250002)
8. [**REPORTING REQUIREMENTS** **36**](#_TOC_250001)
9. [**STATISTICAL METHODOLOGY** **45**](#_TOC_250000)
10. **REFERENCES** **50**
11. **INTRODUCTION**
    1. **Acute Lymphoblastic Leukemia (ALL)**

Significant improvements in outcome for childhood acute lymphoblastic leukemia (ALL) have been observed, with complete response (CR) rates exceeding 90% with modern chemotherapy regimens.1,2 At least 70% of children with ALL can be cured. Adult ALL however has a worse outcome. Expected CR rates are 80% to 90%, and long-term event-free survival (EFS) rates are 20% to 40%.3-7 Prognosis is influenced by age, performance status, organ function, white blood cell count, ALL phenotype (CALLA, T- cell, B-cell, Burkitt's or Burkitt's-like), karyotype [t(1; 19), t(4; 11), Philadelphia (Ph) chromosome], and time to achieve CR.

The longer-term results of hyper-CVAD in adult ALL have been reported8,9. Overall, the group had a median age of 40 years (range 15-90 years); only 20% of the patients were aged 60 or older.9 The majority of patients were considered high-risk for systemic relapse (74%) or central nervous system (CNS) relapse (55%). Compared to the VAD (vincristine, doxorubicin, dexamethasone) program, the outcome with hyper-CVAD was significantly improved with respect to CR rate and overall survival.8,9

The overall CR rate with hyper-CVAD for all subtypes of ALL (n=288) was 92% with an induction mortality of 5% (all due to infections). Eleven percent of the patients required 2 courses to achieve CR; only 3% of the patients failed to achieve a remission with persistence of the leukemia. Central nervous system relapse was low, with a 7% incidence in low-risk patients (given 4 prophylactic intrathecal [IT] treatments), 1% in high-risk patients (16 prophylactic ITs), and 6% in patients with unknown CNS relapse risk (8 prophylactic ITs).9 The 5-year continuous CR rate was 35% with a 5-year survival of 36%. On multivariate analysis, pre-treatment factors associated with a shorter survival included older age, poor performance status, presence of hepatomegaly, high leukocyte count (≥ 50 x 109/L), low platelet count, and Ph-positive disease. When compared to other published standard regimens of MSKCC,5 SWOG,6 Hoelzer et al,4 and Larson et al,7 the outcome was comparable even though the MDACC patients were older and prognostically less favorable.

- 1. **Hyper-CVAD in Ph-positive ALL**

The outcome with hyper-CVAD in newly diagnosed Ph-positive ALL has been reviewed.10 Twenty-nine patients were treated with the regimen from 1992 to 1997. Their median age was 41, and median leukocyte count was 23 x 109/L. The CR rate was 90% with no induction deaths observed. The median survival was 16 months, and median CR duration (CRD) was 10 months. The event-free survival (EFS) rate was 14%. Despite improvements in CR rate and median CRD compared to earlier and other published conventional ALL programs, the 3-year survival and DFS rates were both less than 10%, suggesting that chemotherapy alone may not be sufficient to eradicate the Ph-positive leukemic clone.

- 1. **Tyrosine Kinase Inhibitors in Ph-positive ALL**

Although the single agent response rate with tyrosine kinase inhibitors (TKIs) such as imatinib in relapsed or refractory Ph-positive ALL is encouraging, in one study, ongoing maintained responses (after short follow-up) were observed in only a small number of patients.11 Median estimated time to progression and overall survival for ALL patients were 2.2 and 4.9 months, respectively. Several investigators have conducted in vitro assays to determine the effects of imatinib mesylate in combination with various antileukemic agents such as interferon-alpha, hydroxyurea, daunorubicin, and cytarabine. The combination of imatinib mesylate was additive or synergistic with interferon-alpha, daunorubicin, and cytarabine. Therefore, strategies such as combination imatinib with an effective ALL regimen such as hyper-CVAD have been evaluated.12,13

Thomas et al. have reported on the results of a phase II clinical trial of concurrent hyper- CVAD and imatinib to improve the results of combination chemotherapy.12 Similar studies have been conducted by Ravandi et al. with hyper-CVAD and dasatinib, a more potent breakpoint cluster region-Abelson (BCR-ABL) inhibitor in Ph-positive ALL.14 With the latest updates, both regimens are demonstrating CR rates of 90% and 3- to 5-year survival rates of 40-50%. These studies suggest that addition of a TKI to conventional cytotoxic chemotherapy improves survival compared to either modality alone.

- 1. **Treatment of Ph-positive ALL in Older Patients**

The results remain poor in older patients (age > 60 years) with Ph-positive ALL because of poor tolerance of intensive chemotherapy, mortality with intensive chemotherapy, and lack of access to allogeneic stem cell transplant. Older patients are predisposed to severe toxicity from conventional chemotherapy, which is associated with high mortality rate during consolidation and maintenance despite achieving CR.15 In these patients, induction mortality rate with standard full-intensity chemotherapy was 10% and the death in CR rate was 34%. These patients had a median overall survival (OS) of approximately 15 months and 5-year survival rate of only 20%, which are significantly worse than the outcomes seen in younger patients.

Studies of imatinib or dasatinib in combination with steroids in older patients with Ph- positive ALL have showed encouraging results with high rates of CR.16-18 However, although most older patients are able to achieve CR with these regimens, relapse rates are high with a long-term survival rate of less than 20%. This is likely due to the relatively low complete molecular response (CMR) rate of approximately 19% with these approaches.18 In one study, most of these relapses were due to development of the T315I mutation. This mutation confers pan-resistance to both first and second-generation TKIs.18

- 1. **Low-Intensity Chemotherapy in Older Patients with ALL**

Given the high rate of induction mortality in elderly patients treated with standard-dose intensive induction chemotherapy, some investigators have attempted to de-intensify chemotherapy in order to decrease toxicity and thereby decreasing induction mortality. In elderly patients with Ph-negative ALL, the combination of inotuzumab ozogamycin, an anti-CD22 antibody drug conjugate, with a “mini-hyper-CVD” regimen (in which the anthracycline was excluded and cyclophosphamide, dexamethasone, methotrexate and cytarabine were administered at significantly reduced doses compared to the traditional hyper-CVAD dosing) has shown promising clinical activity with reduced toxicity.19 In the most recent update of these data, 97% of patients achieved CR or CR with inadequate platelet recovery (CRp). The 2-year OS was 70%, which compared favorably to the historical 2-year OS rate of 38% in elderly patients treated with full-intensity hyper-CVAD. These promising results in elderly patients with ALL provide the rationale for combining the mini-hyper-CVD backbone with other novel agents for older patients with ALL, including those with Ph-positive disease.

- 1. **Relapsed/refractory Ph-positive ALL**

Once patients with Ph-positive ALL relapse, outcomes are dismal and there is no clear standard of care. Recent evidence suggests that the bispecific T-cell engaging (BiTE) antibody, blinatumomab, has significant antileukemic efficacy for these patients.20 Nonetheless even with this promising single-agent treatment the overall response rate (ORR) is only 36% and the median OS is approximately 7 months. Clearly, novel treatment options are needed for patients with Ph-positive ALL who are refractory or relapse after initial treatment.

- 1. **Ponatinib**
     1. **General**

AP24534 (United States Adopted Name [USAN]: ponatinib) is a novel 3rd-generation orally-active tyrosine kinase inhibitor (TKI). Ponatinib was specifically developed to inhibit BCR-ABL - the fusion protein that is the product of the Ph chromosome in chronic myeloid leukemia (CML) and in a subset of ALL. It potently inhibits the BCR-ABL protein, as well as mutated forms of the protein that arise in patients that develop resistant to other TKIs. For this reason it is considered a pan-BCR-ABL inhibitor.

The most common single resistant mutation is a transition point mutation at position 944 of the BCR-ABL protein, resulting in a substitution of isoleucine (symbol I) for threonine

(T) at position 315 of the protein designated as T315I.22 The T315I mutation accounts for 15%-20% of all mutants observed in refractory CML. Although dasatinib is effective against some mutations that confer resistance to imatinib therapy, and nilotinib also treats some imatinib-induced mutations, neither is sensitive for T315I. Ponatinib is the only approved TKI that can overcome the resistance conferred by T315I mutation and successfully inhibit the BCR-ABL oncoprotein. Other mutations confer resistance to both

imatinib and dasatinib, such as F317L, while other mutations confer resistance to imatinib and nilotinib, such as E255V. Ponatinib overcomes these mutations as well.

A factor that contributes to therapeutic failure of the first or second generation agents is intolerance to therapy. Although the marketed drugs are convenient and safe, they cause adverse events (AEs) that result in dose reduction or even cessation of therapy in a proportion of patients. The side effect profile of imatinib includes edema, fluid retention, myelosuppression, cardiac toxicity, hepatotoxicity, and hemorrhage. Dasatinib is associated with severe myelosuppression, hemorrhage, fluid retention, effusions, and QT prolongation. Nilotinib is also associated with myelosuppression, QT prolongation, and sudden death. These AEs, and especially fluid retention and myelosuppression, lead to intolerance to continued therapy in a number of patients.

In summary, while the approved therapies for Ph-positive disease represent substantial progress, they fail in a significant proportion of patients because of either the development of resistance or intolerance. Thus, the need for new therapies is evident.

- - 1. **Ponatinib in Ph-positive Leukemias Phase I**

The phase 1 trial of ponatinib enrolled 81 patients with hematologic malignancies who had no other effective treatment options.23 Doses from 2 mg to 60 mg were investigated, and 45 mg was chosen as the recommended phase 2 dose (RP2D). The most common treatment-emergent AEs (TEAEs) (≥30%) were rash (48.1%), nausea (45.7%), abdominal pain (44.4%), fatigue (44.4%), headache (42%), arthralgia (39.5%),

constipation (39.5%), vomiting (38.3%), hypertension (37.0%), peripheral edema (35.8%), platelet count decreased (35.8%), and pyrexia (35.8%). Pancreatitis was the dose-limiting toxicity (DLT) in the phase 1 study, occurring in 11.1% of patients. The serious AEs (SAEs) with the highest incidence (> 5% of patients), regardless of relationship to study treatment, were febrile neutropenia (14.8%), pneumonia (13.6%), pyrexia (12.3%), pancreatitis (11.1%), neoplasm progression (8.6%), abdominal pain (7.4%), atrial fibrillation (7.4%), and dyspnea (6.2%). Serious vascular events occurred in 19 patients (23%): 11 (14%) had cardiovascular SAEs, 4 (5%) had cerebrovascular

SAEs, 4 (5%) had peripheral vascular SAEs, and 2 (2%) had venous SAEs.

**Phase II**

The Phase II clinical trial (PACE) using ponatinib enrolled 449 patients with Ph-positive leukemia, including 32 patients with Ph-positive ALL24. Patients received ponatinib 45 mg orally daily without additional chemotherapy. The majority of patients with Ph-positive ALL had resistance to dasatinib or nilotinib (n = 26, 91%) and 22 patients (69%) had the T315I mutation detected by ABL sequencing. Most of the patients with Ph-positive ALL (n

= 26, 81%) had received two or more TKIs before enrollment. The primary endpoint for the patients with Ph-positive ALL was major hematologic response (MHR), which was

defined as less than 5% blasts on bone marrow morphology and normalization of blood counts. Thirteen patients (41%; 95% CI: 24–59%) with Ph-positive ALL had a MHR and the median time to achieve this was 2.9 weeks (range: 1.6–24). The median duration of response was short at 3 months and the estimated proportion with a sustained response at 12 months was 8%. The MHR response rate from the long-term follow-up data was slightly higher in patients lacking the T315I mutation (50 vs 36%), although the number in each group was small. The complete cytogenetic response rate was 38% (T315I-positive 41% vs T315I-negative 50%). The majority of patients with Ph-positive ALL relapsed after achieving a response (92%, n = 12). The estimated progression-free survival (PFS) and OS at 1 year was 7 and 40%, respectively. Repeat ABL mutation testing was available in 5 of 12 patients after loss of response. All five of these patients had a resistance mutation at baseline and four patients acquired an additional resistance mutation at the time of relapse. No single resistance mutation conferring resistance to ponatinib was identified for the entire cohort. These results demonstrate that ponatinib has activity in relapsed and TKI-resistant Ph-positive ALL, although as a single agent it does not appear to produce durable responses in relapsed patients.

- - 1. **Ponatinib and Hyper-CVAD in Ph-positive ALL**

The combination of ponatinib and hyper-CVAD has been shown to have significant clinical activity as frontline therapy for patients with Ph-positive ALL.25 Thirty-seven patients with newly diagnosed Ph-positive ALL were treated with HCVAD and ponatinib 45 mg daily for the 14 days during induction, then continuously thereafter with possible dose de-escalation to 30 mg and 15 mg daily once a complete cytogenetic response (CCyR) and CMR were obtained, respectively. The CR, CCyR and CMR rates were 100%, 100%, and 74%, respectively. Grade ≥ 3 toxicity included infections during induction (49%), increased LFT’s (35%), thrombotic events (8%), myocardial infarction (8%), skin rash (11%), and pancreatitis (16%). Two potential related deaths from myocardial infarction were observed. This led the investigators to decrease the ponatinib dose based on the depth of a patient’s response (e.g. CR vs. CCyR vs. CMR). With a median follow-up of 33 months, the 3-year overall survival rate is 80%. This compares very favorably to previous experience with either imatinib or dasatinib in combination with hyper-CVAD.12,14 The lower dose schedule (30 mg with further reduction to 15 mg daily) was found to be active, but more importantly safer with no vascular events encountered contrasting with 45 mg daily (15-20% rate of serious vascular events). This is online with data from CML where dose reduction to ponatinib was associated with equivalent efficacy and better safety profile.

- - 1. **Summary**

In summary, the phase 2 clinical trial of hyper-CVAD and ponatinib in patients with Ph- positive ALL has shown significant clinical activity that compares favorably to data from previous trials using imatinib or dasatinib. Given previous experience that full-intensity hyper-CVAD results in unacceptable toxicity in many older patients with ALL, there is a rationale to combine a less intensive chemotherapy backbone with ponatinib in patients

with Ph-positive ALL. Taken together, these studies provide a strong rationale for a phase 2 trial combining ponatinib with blinatumomab in patients with Ph-positive ALL, both in the relapsed/refractory and frontline.

- 1. **Blinatumomab**
     1. **Background**

The Bispecific T-cell Engaging (BiTE) antibody blinatumomab represents the first agent in a novel class that redirects host T-cells to cell surface antigen-expressing cancer cells.

Blinatumomab contains the variable domains of a CD19 antibody and a CD3 antibody that are joined by a non-immunogenic linker. Upon binding to CD19, the cytotoxic T cells become activated and induce cell death via the pore-forming perforin system. This drug was initially administered as an intermittent infusion two to three times weekly, but lack of activity and serious neurologic toxicity caused the schedule of administration to be abandoned. Based on the short half-life of the drug and the mechanism of action, administration as a continuous infusion over several weeks was investigated and this drastically improved the activity of the drug and minimized adverse effects.

- - 1. **Minimal residual disease**

The first study with blinatumomab used as continuous infusion and evaluated its potential role in eradicating MRD.26 MRD-positivity in ALL almost universally heralds systemic relapse and confer a poor prognosis. Patients treated in this study were in hematologic and morphologic CR, but had persistent or reappearing MRD during consolidation chemotherapy. Blinatumomab was given at a dose of 15 mcg/m2/day as a continuous infusion for 28 days every 6 weeks (14 days off). After completing one cycle, responding patients could receive up to three additional consolidation cycles or proceed to allogeneic stem cell transplant (ASCT) if a donor was available. MRD conversion after one cycle was observed in 16 of 20 evaluable patients (80%). In a long-term follow-up update (median observation time 33 months), 12 of the 20 patients remained in CR. The estimated 3-year relapse-free survival was 60%.27 Nine patients underwent ASCT, but interestingly, non-transplanted patients had similar favorable outcome compared to the transplant group. Most relapses after blinatumomab treatment occurred early, within seven months of the start of therapy. Gökbuget et al, assessed single agent blinatumomab in 116 patients with ALL in CR but with MRD positivity. Most patients had

≥ 3 courses of chemotherapy and ≥ 35% were in CR2. Blinatumomab was given at 15 mcg/m2/day continuous infusion for 28 days every 6 weeks for 4 cycles. Approximately 78% achieved MRD negativity after one cycle and 80% after 4 cycles.28 With a median follow-up of 29 months, the median OS was 36 months and the RFS was 19 months. The median OS for those who achieved MRD negative status was 40 months versus 12 months for those who remained MRD positive. Notably, allo-SCT did not confer a survival benefit for patients who achieved MRD negativity in first remission. These results provide evidence that a strategy of MRD-directed therapy using monoclonal antibodies is useful in improving outcomes in ALL.

- - 1. **Relapsed and refractory disease**

Blinatumomab was subsequently studied in patients with active systemic ALL relapse.29 Three dose levels were explored, all involving blinatumomab administration as a continuous infusion for 28 days every 6 weeks. The overall response rate (CR or CR with incomplete count recovery) within two cycles of therapy was 68%. The estimated median survival was 9 months.29 The final dose selected for future studies was 5 mcg/m2/day during week one, and 15 mcg/m2/day during the following three weeks. In a confirmatory open-label, single-arm, multicenter phase II study in 189 patients with relapsed-refractory disease, the overall response rate was 43% with 80% of the responses occurring within cycle 1. The median response duration and overall survival were 9 and 6 months, respectively. A Phase III randomized trial (TOWER study) compared blinatumomab to investigator’s choice chemotherapy in patients with relapsed/refractory ALL. OS was the primary endpoint, and duration of CR, CR, and MRD negativity were secondary endpoints. Blinatumomab improved response rates and overall survival compared to SOC (7.7 months vs 4.0 months; p=0.012).

- - 1. **Philadelphia-positive ALL**

Blinatumomab was also evaluated in Phase II ALCANTRA trial in patients with relapsed/refractory Ph-positive ALL. Single agent blinatumomab was given at standard dose for up to 5 cycles in 45 patients; after the first two cycles, 36% achieved CR or partial hematologic response. With a median follow-up of 9 months, the median RFS and OS were 6.7 months and 7.1 months, respectively. Of 16 total responders, the minimal residual disease (MRD) negativity rate was 88%, and 44% of patients were able to receive allo-SCT.

- 1. **Rationale for combining ponatinib and blinatumomab**

The current data with ponatinib suggests it to be the best TKI in patients with Ph-positive ALL. Studies comparing ponatinib to dasatinib in combination with chemotherapy established the superiority of ponatinib with at least 20 percent improvement in the rates of complete molecular response and 3-years event-free and overall survival. Despite the high efficacy of TKI, when used as single agent, the rates of CMR are low (20% ranges) and responses are not durable. Therefore combination of chemotherapy and TKI is superior to TKI alone. Blinatumomab has shown significant activity in Ph-positive ALL and was found to be superior to chemotherapy in randomized trial in the relapse/refractory setting. Furthermore, immunotherapy with blinatumomab seems to have minimal myelosuppression and is well tolerated relative to traditional cytotoxic therapy. Hence combining blinatumomab with ponatinib warrants further investigation. If positive, such combination constitutes a practice changing trial and may become a new standard of care of patients with de novo Ph-positive ALL.

- 1. **MDACC preliminary experience using the combination of blinatumomab and ponatinib**

We assessed the combination of blinatumomab and tyrosine kinase inhibitors in 11 patients with relapsed/refractory Ph+ ALL (n=8) and chronic myeloid leukemia in blast crisis (n=3). These patients were treated with the combination blinatumomab and a TKI (ponatinib 30 mg daily, n=8; Dasatinib 100 mg daily, n=2; bosutinib 400 mg daily, n=1). All patients have previously failed at least one line of chemotherapy, including allogeneic stem cell transplantation, and one class of TKIs. Patients were treated for either overt hematological relapse (n=6) or persistent MRD following other regimens (n=5).

The complete hematologic, cytogenetic and molecular response rates were 50% (3/6), 71% (5/7), and 60% (6/10), respectively. Three cases of grade 2 cytokine release syndrome were observed, all of which resolved with steroids and tocilizumab. No cardiovascular adverse events were encountered. With a median follow-up of 5 months, the median survival was not reached; the 6-month overall survival rate was 70%. (Manuscript submitted for publication)

- 1. **Rational of the eligibility for including previously untreated ≥ 18 years of age patients**

The protocol is currently open for frontline therapy for 60 years and older. The patients treated so far have responded so well with 5 out of 6 patients achieving a complete molecular remission. Furthermore, studies done in Europe have shown the safety and efficacy of this regimen with TKI in the younger population. In Italy, 63 patients were treated with a median age of 44 years. The treatment was found to be safe with a CMR rate of 55%. Therefore, we plan to amend this study to allow adult patients 18 years and older to be enrolled in this study. Currently this study is open for patients unfit for intensive chemotherapy or 60 years and older. Inclusion criteria modified to include 18 years and older.

- 1. **Update of the experience as of October 9, 2021.**

We would like to increase the sample size from 60 patients to 90 patients. This study has so far accrued around 50 pts. The results are very promising with CMR rate of 85% in the frontline with 2 years survival surpassing 95%. This is very encouraging results. We would like to expand the study and be able to confirm if it can constitute a new standard of care. Furthermore, we will look for predicted factor for outcome and further analysis that can consolidate the role of blinatumomab + ponatinib as standard of care for Ph+ ALL.

- 1. **Update of the experience as of March 15, 2023.**

As of March 15th, 2023, 83 patients were enrolled. 66 patients enrolled in a front-line and 17 in the relapsed cohort. The results so far are very promising with a 3 years survival of

95% in the front-line cohort. These results are practice changing. This can become a new standard of care. Therefore, we aim to amend this study to expand it to 120 patients by adding 30 additional patients. As of March 15th, 2023, 83 patients were enrolled. 66 patients enrolled in the frontline cohort and 17 in the relapsed cohort. The results so far are very promising with a 3 years survival of 95% in the frontline cohort. These results are practice changing. This can become a new standard of care. It will be very important to analyze more precisely the benefits in Ph-positive ALL subsets, e.g. P190 vs P210; Achievement of PCR/NGS MRD negativity vs not, age < 60 vs> 60, CG Ph only vs additional abnormalities, etc. This will allow us to confirm the findings already obtained and with the longer follow up, this will allow us to have a multivariate-analysis done to identify patients who will benefit from this strategy, such a strategy can become the new standard of care. Therefore, we would like to expand the study to add 30 additional patients.

- 1. **Update of the experience as of April 4, 2023.**

We have treated 84 patients on the study, among them 67 patients in the frontline. Within the past of 18 months we started seeing CNS relapses. Overall, we have 3 CNS relapses. This could be due to the fact that we eliminated chemotherapy completely.

Essentially, we eliminated cytarabine and high dose methotrexate known to penetrate CNS barrier.

Similarly, in the Italian group in the D-ALBA trial have seen 5 CNS relapses and that triggered them to increase the number of intrathecal chemotherapies. Therefore, in the long run to avoid CNS relapses, we would like to increase the number of intrathecal from 12 to 15. These will be done 3 LP per cycle. We amend the study accordingly.

- 1. **Update of the methotrexate and cytarabine experience as of September 27, 2023.**

As of today, 62 patients are evaluable the 3-year survival is 89%. We observed 4 CNS relapses. This is the rate of 6.5% which is significantly higher than the historical relapses on HCVAD protocol. This could be due to the lack of systemic chemotherapy with CNS penetration mainly cytarabine and methotrexate. This was mainly observed in patients with high WBC at baseline and baseline CNS disease. By multivariate analysis performed to evaluate baseline factors associated with CNS relapse, increase WBC ≥ 75K/uL was associated with a higher likelihood of CNS relapse (OR=10.332; p=0.047). Therefore, we plan to amend the treatment and for patients at high risk for CNS relapse (high WBC and CNS disease at baseline), we will administer 2 cycles of high dose methotrexate (500 mg/m2 on D1 given as 100 mg/m2 over 2 hours and 400 mg/m2 over 22 hours; this dose will be reduced to 250 mg/m2 for patients 60 years and older given as 50 mg/m2 over 2 hours and 200 mg/m2 over 22 hours) and high dose cytarabine (1 g/m2 q12 hours on D2- 3; this dose will be reduced to 500 mg/m2 for patients 60 years and older) and blinatumomab starting on D4 till D21. This will be recycled on D28 and 2 cycles will be given. Ponatinib will be continued and IT will be administered as per institutional standard to complete a total of 15 (2 ITs per cycle during the systemic cycles and 3 ITs during

blinatumomab only cycles). Afterwards patients will resume the treatment with blinatumomab and ponatinib for 2 additional cycles.

- 1. **Update of experience of as of January 11, 2024.**

Originally in - protocol, blinatumomab and ponatinib were to be given up to 4 cycles in patient with MRD relapsed where MRD was noted by PCR 0.01%. The amendment v25 is to add that for any MRD positivity by NGS or PCR or flow cytometry, patients can receive blinatumomab. The reason is so far with 96 treated patients, patients with NGS positivity ultimately relapsed therefore early intervention can lead to better outcome.

Therefore, amending the study to allow any patient with any NGS positivity to be treated with blinatumomab and to define MRD positive disease.

1. **STUDY OBJECTIVES**

**2.1. Primary Objective:**

To evaluate the complete molecular response rate in cohort 1 (newly diagnosed Ph- positive and/or BCR-ABL-positive ALL) and the overall response (CR+CRi) rate in cohort 2 (relapsed/refractory disease).

- 1. **Secondary Objective**:

To evaluate other clinical efficacy endpoints (complete cytogenetic response, complete molecular response, event-free survival and overall survival) and safety of the regimen.

- 1. **Exploratory Objectives:**
     - To characterize the role of *ABL1* kinase domain mutations on treatment failure and relapse in patients with Ph+ ALL treated with blinatumomab and ponatinib
     - To determine the impact of recurrent genomic alterations at diagnosis on RFS in patients with Ph+ ALL treated with blinatumomab plus ponatinib
     - To investigate the impact of next-generation sequencing-based minimal residual disease assessment on relapse-free survival in patients with Ph+ ALL.
     - To determine the effect on immune cell subsets in patients with Ph+ ALL treated with blinatumomab plus ponatinib.

1. **SELECTION OF PATIENTS**

Patients will be selected from those referred to the Leukemia department at M D Anderson Cancer Center through the normal of process of referral. Eligible patients will be registered after the process of consenting on the MD Anderson protocol and data monitoring system.

- 1. **Inclusion Criteria**

1. Diagnosis of one of the following:
   1. Patients ≥ 18 years of age with previously untreated Ph-positive ALL [either t(9;22) and/or BCR-ABL positive] (includes patients initiated on first course of therapy before cytogenetics known) or with lymphoid accelerated or blast phase CML. These patients could have received one or two courses of chemotherapy with or without other TKIs and still eligible. (Patients with lymphoid accelerated or blast phase CML will be evaluated separately)
      1. If they achieved CR, they are assessable only for event-free and overall survival, *or*
      2. If they failed to achieve CR, they are assessable for CR, event-free, and overall survival
   2. Patients ≥ 18 years of age with relapsed/refractory Ph-positive ALL or with previously treated lymphoid accelerated or blast phase CML (Patients with lymphoid accelerated or blast phase CML will be evaluated separately)
   3. Patients ≥ 18 years of age with ALL MRD positive (either by NGS or PCR or flowcytometry) or with previously treated lymphoid accelerated or blast phase CML (Patients with lymphoid accelerated or blast phase CML will be evaluated separately)
2. Performance status ≤2 (ECOG Scale)
3. Adequate liver function as defined by the following criteria (unless the increased values are judged to be leukemia disease related):
   1. Total serum bilirubin < 2 x upper limit of normal (ULN), unless due to Gilbert’s syndrome
   2. Alanine aminotransferase (ALT) < 3 x ULN, OR
   3. Aspartate aminotransferase (AST) < 3 x ULN
4. Adequate pancreatic function as defined by the following criteria:
   1. Serum lipase and amylase < 1.5 x ULN
5. For females of childbearing potential, a negative urine pregnancy test must be documented
6. Female patients who:

- Are postmenopausal for at least 1 year before the screening visit, OR
- Are surgically sterile, OR
- If they are of childbearing potential, agree to practice 2 effective methods of contraception, at the same time, from the time of signing the informed consent through 4 months after the last dose of study drug, or agree to completely abstain from heterosexual intercourse

1. Male patients, even if surgically sterilized (i.e., status post-vasectomy), who:

- Agree to practice effective barrier contraception during the entire study treatment period and through 4 months after the last dose of study drug, or
- Agree to completely abstain from heterosexual intercourse

1. Adequate cardiac function as assessed clinically by history and physical examination.
2. Signed informed consent
   1. **Exclusion Criteria**
3. Active serious infection not controlled by oral or intravenous antibiotics.
4. History of acute pancreatitis within 1 year of study or history of chronic pancreatitis
5. History of alcohol abuse
6. Uncontrolled hypertriglyceridemia (triglycerides > 650mg/L)
7. Active secondary malignancy other than skin cancer (e.g., basal cell carcinoma or squamous cell carcinoma) that in the investigator’s opinion will shorten survival to less than 1 year.
8. Active Grade III-V cardiac failure as defined by the New York Heart Association Criteria.
9. Uncontrolled, or active cardiovascular disease, specifically including, but not restricted to:
   - Myocardial infarction (MI), stroke, or revascularization within 3 months
   - Unstable angina or transient ischemic attack
   - Congestive heart failure prior to enrollment, or left ventricular ejection fraction (LVEF) less than lower limit of normal per local institutional standards prior to enrollment
   - Diagnosed or suspected congenital long QT syndrome
   - Clinically significant atrial or ventricular arrhythmias (such as artrial fibrillation, ventricular tachycardia, ventricular fibrillation, or Torsades de pointes) as determined by the treating physician
   - Prolonged QTc interval on pre-entry electrocardiogram (> 470 msec) unless corrected after electrolyte replacement or approved by cardiologist
   - Significant venous or arterial thromboembolism including deep venous thrombosis or pulmonary embolism. Patients with a history of treated prior superficial or catheter associated will not be considered as significant embolism and after discussion with PI will not be excluded from eligibility.
   - Uncontrolled hypertension (diastolic blood pressure >90mmHg; systolic

>140mmHg). Patients with hypertension should be under treatment on study entry to effect blood pressure control

1. History or presence of clinically relevant CNS pathology or event such as epilepsy, childhood or adult seizure, paresis, aphasia, stroke, severe brain injuries, dementia, Parkinson’s disease, cerebellar disease, organic brain syndrome, psychosis or severe (grade 3 or above) CNS events including ICANS from prior CART or other T cell engager therapies. Patient with active CNS leukemia - will NOT be excluded
2. Current autoimmune disease or history of autoimmune disease with potential CNS involvement
3. Treatment with any investigational antileukemic agents or chemotherapy agents within 2 weeks prior to study entry, unless full recovery from side effects has occurred or patient has rapidly progressive disease judged to be life-threatening by the investigator.
4. Pregnant and lactating women will not be eligible; women of childbearing potential should have a negative pregnancy test prior to entering on the study and be willing to practice methods of contraception. Women do not have childbearing potential if they have had a hysterectomy or are postmenopausal without menses for 12 months. In addition, men enrolled on this study should understand the risks to any sexual partner of childbearing potential and should practice an effective method of birth control.
5. History of significant bleeding disorder unrelated to cancer, including:
   - Diagnosed congenital bleeding disorders (e.g., von Willebrand’s disease)
   - Diagnosed acquired bleeding disorder within one year (e.g., acquired anti-factor VIII antibodies)
6. Patients with documented significant pleural or pericardial effusions unless they are thought to be secondary to their leukemia.
7. Known active infection with HIV, HBV, HCV.
8. **TREATMENT OF SUBJECTS**
   1. All patients will be consented and registered in OnCore. Subjects’ concomitant medications will be collected and readily available in the MDACC electronic health record and will not be entered into the CRF.

Induction and consolidation therapy will be performed at MD Anderson Cancer Center. Following consolidation and maintenance therapy may be done at MD Anderson or under the care of the local oncologist with supervision by MD Anderson Leukemia physician.

Patients must be discontinued from the trial in the event of myocardial infarction or stroke, or for development or progression of arterial disease necessitating revascularization.

Variations in dose reductions or the administration of ponatinib and/or blinatumomab, or chemotherapy treatment sequence modification of methotrexate and/or cytarabine (such as dose reductions or omission), or supportive care dose schedules other than those suggested below are allowed in the best interest of patients. Such patients should be discussed with the principal investigator. Dose escalations of chemotherapy above those outlined in the protocol; however, are not allowed.

Examples of these clinical scenarios include:

1. Treatment delays (> 14 days from recovery) despite hematologic recovery for reasons of patients request or unavoidable social situations. Treatment delays to allow recovery from infections or other toxicities of therapy will not be considered deviations, as these are expected complications of the therapy.
2. Dose reductions or alterations in the chemotherapy administration including treatment delays beyond those specified in the protocol for reasons of patient request or unavoidable social situations or logistic situations such as travel, inclement weather, scheduling issues, etc. will not be considered deviations. Dose reductions performed for clinical reasons will not be considered deviations, as patients may have unique toxicities or tolerance not accounted for by standard dose reductions.
3. Other clinical scenarios after approval by the Principal Investigator including dose modification of chemotherapy treatment and sequence such as dose reductions, schedules, or omissions of blinatumomab, ponatinib, methotrexate, cytarabine are allowed in the best interest of patients.

Every effort will be made to adhere to the schedule of events and all protocol requirements. Variations in schedule of events and other protocol requirements that do not affect the rights and safety of the patient will not be considered as deviations. Such variations may include laboratory assessments completed outside of schedule. All dose adjustments will be made according to the protocol unless otherwise specified.

1. When necessary, after approval by the Principal Investigator, investigational drug may be shipped directly to the patient for reasons of patient request or unavoidable social situations or logistic situations such as travel, inclement weather, scheduling issues, other unexpected situations, etc.
   1. **General Considerations (Figure 1)**
2. Patients with newly diagnosed, active disease who are aged 60 years or older may receive the first induction course in the protective environment unless they refuse, are unable to be confined, and/or medical illness prohibits. Protective environment is optional.
3. Tumor lysis syndrome will be managed per institutional standard of care.
4. CNS prophylaxis: total number of prophylactic intrathecal treatments will be 15. A total of 3 will be given with each course of blinatumomab on day 1 (+/- 3 days), day 15 (+/- 3 days) and day 29 (+/- 3 days) until total number reached. Intrathecal therapy will consist of Methotrexate 12mg (6mg if intra-ommaya) and alternate with Cytarabine 100mg. Missed intrathecal can be made up. Intrathecal route via lumbar puncture as opposed to intra-ommaya is preferred. Patients with CNS leukemia will be treated with therapeutic intrathecal therapy as per standard of care. This usually consists of twice weekly intrathecal until CSF negative for at least twice then weekly intrathecal for 2 months, then intrathecal every other week for 2 months, then intrathecals monthly to complete a total of 1 year of therapy. IT chemotherapy can be delayed in case of high white count and/or if felt to be in the best interest of the patient and after discussion with the PI. After patient has completed the combination therapy, patient will have surveillance and monitoring lumbar puncture as standard of care without intrathecal chemotherapy every 3 months (± 1 month) for 2 years of the completion date.
5. Blinatumomab will be administered as continuous intravenous infusion at a dose of 28 μg/24 hours over 4 weeks followed by a treatment-free period of 2 weeks, defined as one 6-week treatment cycle for up to 5 cycles (one induction and up to 4 consolidations) for patients with WBC < 75K/uL and for patients with WBC ≥ 75K/uL and patients with CNS disease at baseline on cycles 1-2 and 5-6, and additional up to 4 cycle during maintenance if PCR > 0.01%. Ideally, we will start blinatumomab when peripheral blood blasts are ≤5.0 x 109/L. Refer to sec. 4.3.1.
6. Ponatinib will be administered as continuous therapy through induction and consolidation therapy with blinatumomab, and infinitely as maintenance therapy thereafter.
7. Patients who do not complete all therapy per protocol, but remain in remission, will not be considered off-protocol, but will be monitored for disease-free survival only. An end-of-treatment date will indicate completion of therapy.
8. Variations in infusion times due to minor differences in IV bag overfill/underfill and institutional procedure on flushing chemotherapy lines will not result in protocol deviation. All infusion times are considered approximate.
9. Prophylactic antibiotics may be given with each course until neutrophil recovery to 500/μL or greater (or other antibiotics if being treated for active infection). Suggestions include: Levaquin 500 mg po daily (or other quinolone) or trimethoprim- sulfamethoxazole (or other appropriate antibacterial agent such as cefpodoxime 200 mg BID. Fluconazole 400mg po daily or other appropriate antifungal agent. Valacyclovir 500 mg PO daily or acyclovir 400mg PO BID or other appropriate antiviral agent.
10. Patients should receive treatment with a statin, as prophylaxis against arterial thrombotic events, if indicated. Rosuvastatin, 5-10 mg per day, is suggested.
11. Patients should receive low-dose aspirin (e.g.75-100mg/day), as prophylaxis against arterial thrombotic events, if indicated. In general, aspirin should be held for platelets

<50,000units/mcL or if more strict parameters are deemed appropriate per treating physician discretion.

1. If one study drug is discontinued for any reason, patient may continue with the other study drug on protocol after discussion with the primary investigator.

**Figure 1.**


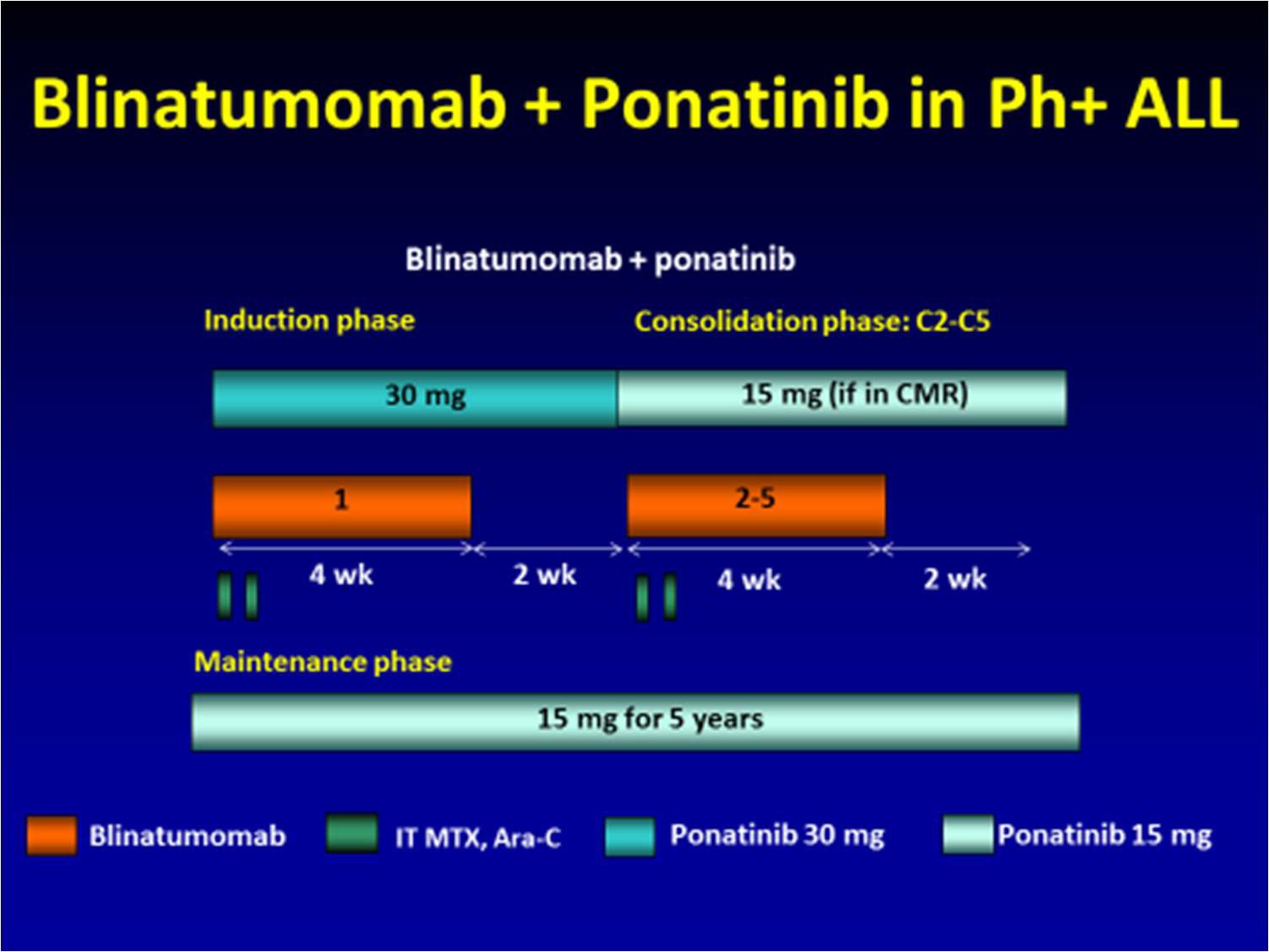


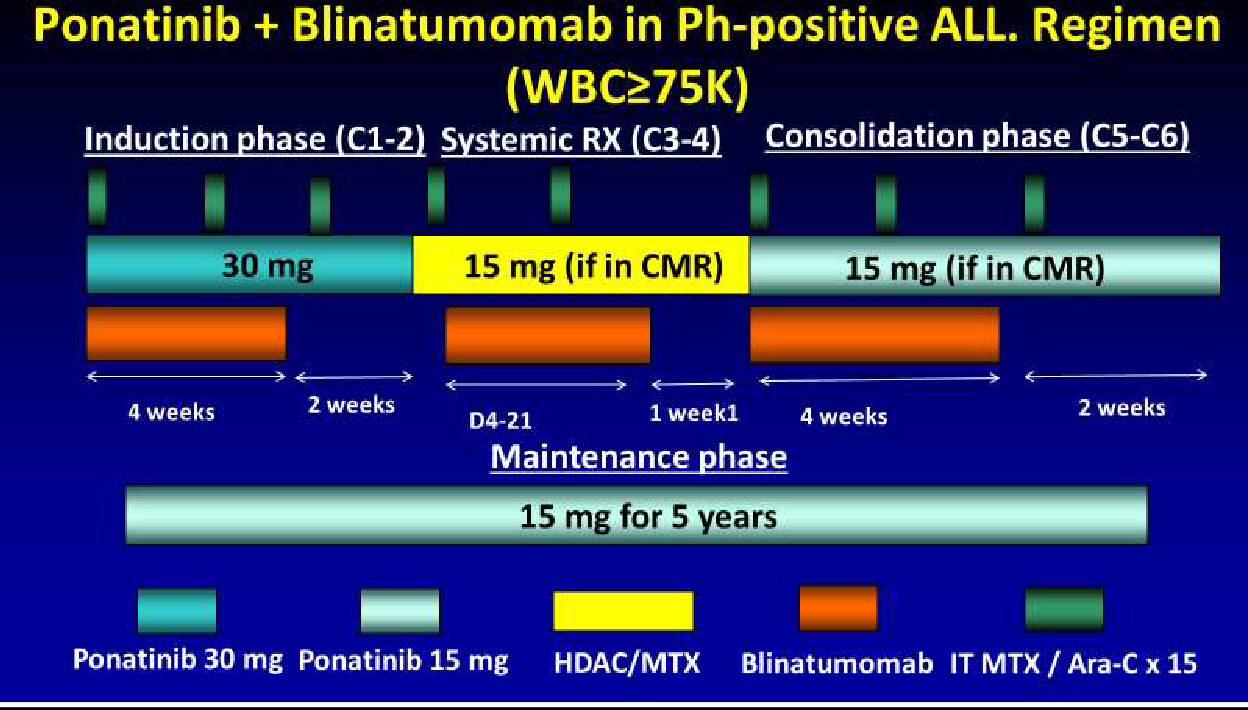
**For patients with WBC<75K/uL: cycles 1-5 and for patients with WBC ≥ 75K/uL and patients with CNS disease at baseline, cycles 1, 2, 5 and 6 – Blinatumomab + ponatinib**

1. Blinatumomab will be administered as continuous intravenous infusion at a dose of 28 μg/24 hours over 4 weeks followed by a treatment-free period of 2 weeks, defined as one 6-week treatment cycle for 4 cycles. In the first cycle, the initial dose of blinatumomab will be 9 μg/day for the first 2 days of treatment (to mitigate for potential cytokine release syndrome (CRS) and CNS events associated with introduction to blinatumomab) which then will be escalated (dose step) to 28 μg/day starting on Day 3 through Day 28. For all subsequent cycles, 28 μg/day will be the dose for all 4 weeks of continuous treatment and follow by a 2 weeks treatment-free interval every 6 weeks.
2. During cycle 1, Ponatinib will be given as 30mg PO daily continuously (may shift ± 4- 5 days if therapy changed once results of cytogenetics known).

For subsequent courses ponatinib will be administered as 30mg PO daily continuously without interruptions unless due to toxicity. Beginning in cycle 2, in patients who achieved complete molecular response (CMR), ponatinib should be further reduced to 15 mg per day unless judged necessary to stay on 30 mg and after discussion with the PI.

1. CNS prophylaxis a total of 3 will be given with each course of blinatumomab on day 1 (+/- 3 days), day 15 (+/- 3 days) and day 29 (+/- 3 days) until total number of 15 intrathecal reached. Intrathecal therapy will consist of Methotrexate 12mg (6mg if intra- ommaya) and alternate with Cytarabine 100mg. Missed intrathecal can be made up.

**For patients with WBC≥75K/uL and patients with CNS disease at baseline, Cycles 3 and 4 – Metrotrexate (MTX)+cytarabine+blinatumomab+ponatinib**

1. Methotrexate (MTX) 500 mg/m2 IV given as 100 mg/m2 IV approximately over 2 hrs (± 1 hour) followed by 400 mg/m2 for approximately over 22 hrs (± 2 hour) on **Day 1. For ≥ 60 years old patients**, this dosed will be reduced to Methotrexate (MTX) 250 mg/m2 IV given as 50 mg/m2 IV approximately over 2 hrs (± 1 hour) followed by 200 mg/m2 for approximately over 22 hrs (± 2 hour) on **Day 1**.

Total duration of administration is 24 hours (2 plus 22 hours) (± 3 hours).

1. Cytarabine 1 g/m2 IV approximately over 2 hrs (± 1 hour) for approximately every 12 hrs for 4 doses on **Days 2 and 3**. Reduce to 0.5 g/m2 IV approximately over 2 hrs (± 1 hour) every 12 hrs for 4 doses on days 2 and 3 for:
   - Neurotoxicity (Grade 2 reversible cerebellar toxicity or other ara-C related CNS toxicity) with previous courses.
   - Creatinine ≥ 1.5 mg/dL.
   - MTX > 20 mcM at time "0" (see below), confirmed on repeat sample.

**For ≥ 60 years patients**, this dosed will be reduced to Cytarabine 0.5 g/m2 IV approximately over 2 hrs (± 1 hour) approximately every 12 hrs for 4 doses on **Days 2 and 3**.

1. Leucovorin rescue 50 mg IV followed by 15 mg IV every 6 hours beginning 12 hrs (± 3 hours) post MTX completion for approximately 8 doses until achieved MTX clearance level. Additional rescue allowed as indicated for elevated levels or delayed methotrexate clearance.
   - Check MTX around time 0h, 24h, and 48h post completion of MTX unless cleared (e.g. 0.15 mcM or less).
   - if > 20 mcM at time "0", hold ara-C and repeat level; if continues to be > 20 mcM reduce ara-C to 0.5 g/m2 (or 50% of original dose whichever is lower) IV approximately over 2 hrs (± 1 hour) for approximately every 12 hrs for 4 doses on days 2, 3.
   - if > 1 mcM at 24 hrs or > 0.1 mcM at 48 hrs, increase citrovorum rescue until serum MTX level is < 0.1 mcM.
   - Clearance to levels 0.15 mcM or less is acceptable in patients with normal renal function.
2. Consider oral acetazolamide 250 mg PO twice daily to promote MTX excretion if the urine pH is <7.0.
3. Blinatumomab 28 μg/day on **Days 4 - 21** of continuous treatment followed by 1 week treatment-free interval.
4. In patients who achieved complete molecular response (CMR), ponatinib should be further reduced to 15 mg per day unless judged necessary to stay on 30 mg and after discussion with the PI.
5. Pegfilgrastim product 6 mg as a one-time dose or filgrastim product (G-CSF) 10 mcg/kg (rounded) subcutaneously daily (or 5 mcg /kg twice daily) starting after completion of cytarabine doses until post-nadir granulocytes greater than or equal to

1.0 x 109/L.

1. CNS prophylaxis: Methotrexate 12 mg intrathecally (6 mg via Ommaya reservoir) Day 8 (±3 days). Cytarabine 100 mg intrathecally Day 2 (± 3 days).

Suggested Dose Modifications:

1. Cytarabine:
   1. 0.5 g/m2 IV over 2 hrs (± 1 hour) every 12 hrs for 4 doses on days 2, 3:
      - Creatinine ≥ 1.5 mg/dL.
      - Time "0" MTX level > 20 mcM (on repeat level).
      - Age ≥ 60 years.
      - Grade 2 reversible cerebellar toxicity related to high-dose Ara-C.
   2. 0.5 g/m2 IV continuous infusion days 2, 3:
      - Grade 2 reversible cerebellar toxicity related to ara-C 0.5 g/m2 over 2 hours or grade 3 reversible cerebellar toxicity related to any dose of ara-C.
2. Methotrexate:
   1. 25% to 50% reduction:
      - Grade 3 or worse mucositis with previous methotrexate course.
   2. 25% to 75% reduction:
      - delayed excretion and/or nephrotoxicity with previous methotrexate course.
   3. 50% reduction:
      - pleural effusion or ascites (drain effusion if possible)
      - Calculated creatinine clearance:
      - 10 to 50 ml/min: reduce by 50%
      - < 10 ml/min: hold MTX
      - Age ≥ 60 years.
   4. **Blinatumomab**

Blinatumomab will be supplied by Amgen as 3 mL single-use glass injection vials containing a sterile, preservative-free, white to off-white, lyophilized powder for intravenous (IV) administration following reconstitution with sterile water for injection (sWFI). Each vial contains a target of 38.5 μg blinatumomab (nominal) formulated with 25 mM citric acid monohydrate 15% (W/V) trehalose dihydrate, 200 mM lysine hydrochloride and 0.1% (W/V), polysorbate 80, pH 7.

Since blinatumomab will be administered via continuous intravenous route, it needs to be stabilized at low concentrations to prevent absorption to surfaces. Therefore, the IV bag must be conditioned by prior addition of a product-specific diluent (IV solution stabilizer), resulting in a final diluent concentration of 0.5 mM citrate, 25 mM lysine hydrochloride and 0.002% (w/v) polysorbate 80. Sterile water for injection and supplies required for reconstitution and injection of blinatumomab will not be provided to clinical sites.

- - 1. **Blinatumomab dosage, administration, and schedule**

Blinatumomab is administered as a continuous intravenous infusion (CIVI). For patients with WBC < 75K/uL - cycles 1-6 and for patients with WBC ≥ 75K/uL and patients with CNS disease at baseline - cycles 1, 2, 5 and 6, a single cycle of blinatumomab treatment is 6 weeks in duration, which includes 4 weeks of blinatumomab CIVI followed by a 2 week treatment-free interval. The treatment-free interval may be prolonged by up to 7 days, if deemed necessary by the investigator. In the first induction cycle, the initial dose of blinatumomab will be 9 μg/day for the first 4 days of treatment (to mitigate for potential CRS and CNS events associated with introduction to blinatumomab) which then will be escalated (dose step) to 28 μg/day starting on day 5 through day 28 (week 4). For patients with WBC < 75K/uL all subsequent cycles and patients with WBC ≥ 75K/uL and patients with CNS disease at baseline cycles 1-2 and 5-6, blinatumomab 28 μg/day will be the dose for all 4 weeks of continuous treatment. For patients with WBC ≥ 75K/uL and patients with CNS disease at baseline cycles 3-4, blinatumomab 28 μg/day will be the dose on Days 4-21 of continuous treatment

Patients who weigh less than 45kg at the start of each Blinatumomab cycle will have blinatumomab dosing discussed and approved by the principle investigator.

The drug administration should not be interrupted, if possible. In case of infusion interruption, due to any technical or logistic reason, the interruption should be as short as possible and the infusion continued at the earliest time possible. Every interruption longer than 1 hour should be documented. If the interruption is longer than 4 hours, re-start of the infusion should be performed in the hospital, under the supervision of the investigator. The dose to be resumed will be discussed with the investigator. The subject should be observed overnight for possible side effects after the re-start. Administration of dexamethasone premedication as described in **Table A** is recommended. If possible, the infusion duration before and after an interruption should total 28 days per treatment cycle and follow by a 2 weeks treatment-free interval.

- - 1. **Blinatumomab inpatient dosing**

It is recommended that subjects are hospitalized at least during the first 6 days of the first induction cycle and the first 2 days of cycle 2. Close monitoring during the first 48 hours of treatment in the first 2 cycles will be indicated because of the potential adverse events associated with T-cell redistribution and potential cytokine release effects triggered by the administration of blinatumomab.

- - 1. **Blinatumomab outpatient dosing**

After a patient meets the minimum criteria for inpatient administration and monitoring as described in the above section, and if a subject is deemed stable by the investigator, continuation of blinatumomab infusion may continue as an outpatient.

In the outpatient setting, the subject will return to the study site for changes of infusion bags.

In the event of drug interruptions of >4 hours, the restart of the infusion should be performed in the clinic/hospital under the supervision of the investigator.

- - 1. **Dexamethasone premedication**

Premedication with dexamethasone is intended to prevent CRS events associated with blinatumomab treatment. Within 1 hour before the start of treatment in each treatment cycle and/or dose escalation, mandatory premedication with dexamethasone at 20 mg IV is required for the prevention of CRS resulting from blinatumomab.

Patient with hyperleukocytosis and blasts >5000, may receive dexamethasone 40 mg daily for up to 4 days before starting blinatumomab. Patient with high tumor load can have side effects on blinatumomab mainly cytokine release syndrome. The administration of dexamethasone will allow to reduce rapidly the count and avoid and decrease the rate of cytokine release syndrome

- - 1. **Dose modifications (interruptions, withholdings, and criteria for restarting treatment)**
       1. **Infusion interruption/dose modification due to adverse events**

Common Terminology Criteria for Adverse Events (CTCAE) grade 4 adverse events at least possibly related to blinatumomab will require permanent discontinuation of blinatumomab. For CTCAE grade 4 adverse events that are numerically defined laboratory parameters, independent investigator assessment should be used to determine the risk and benefit for each individual patient to continue or discontinue blinatumomab treatment.

CTCAE grade 3 cytokine release syndrome, and CTCAE grade 3 DIC/coagulopathy treatment with blinatumomab will be interrupted until the event

resolves to at least grade 1. Blinatumomab can then be restarted at the lowest starting dose (9 μg/d). If the AE lasts for ≥ 2 weeks, then blinatumomab will be permanently discontinued.

For all other CTCAE grade 3 events and clinically significant laboratory value changes, investigator assessment should be used to determine the risk and benefit to continue blinatumomab therapy with or without dose reduction or discontinue therapy.

Patients who have been dose reduced will have an option to receive the higher dose level once the AE has resolved to grade 1 or less for at least 7 days. Re-start of the infusion should be performed in the hospital. Before blinatumomab is re-started, premedication with dexamethasone 20 mg IV is required. The patient should be observed overnight for possible side effects after the restart.

In addition to the events described above, the dose may be temporarily or permanently reduced to 9 μg/day if, by investigator´s judgment, it is necessary for safety reasons.

After at least 7 days of dosing at 9 μg/day, the dose may be increased to 28 μg/day or treatment may be continued at the dose of 9 μg/day after consultation with the investigator.

If the interruption after an adverse event is no longer than 7 days, the same cycle will be continued. The infusion duration, before and after an interruption, should total 28 days per treatment cycle. If an interruption due to an adverse event is longer than 7 days, a new cycle will start. In addition, an incomplete treatment cycle with a treatment duration of less than 2 weeks will have to be repeated (eg, if cycle 1 was interrupted on day 8 for more than 7 days, the next cycle will be denoted as cycle 1.1 and the same assessments will be performed as in cycle 1). For cycle 1.1, subjects will be started at 9 μg/day for the first 7 days of dosing followed by a dose step to 28 μg/day beginning at day 8 and continuing for the remainder of cycle 1.

An infusion interruption of more than 2 weeks due to an adverse event related to blinatumomab will lead to permanent discontinuation of treatment. In case of logistical difficulties, restart of treatment can be postponed for up to 7 additional days without resulting in permanent treatment discontinuation.

Treatment may be also interrupted or permanently discontinued at the discretion of the investigator if any clinical/laboratory adverse event is considered to be medically relevant.

In case of signs of cytokine release, dexamethasone should be administered orally or IV at a dose of at maximum 8 mg every 8 hours for up to 3 days. The dose can then be reduced step-wise over 4 days.

- - - 1. **Infusion interruption/dose modification due to CNS events**

In case of CNS-related adverse events, dexamethasone should be administered at a dose of at least 24 mg/day. The dexamethasone dose will then be reduced step-wise over 4 days.

In case of CTCAE grade 3 or higher CNS-related adverse events, blinatumomab will be stopped immediately and a physical exam, vital signs and safety laboratory tests will be performed. Additional measures can be taken upon discretion of the investigator, depending on the nature of the adverse event. Diagnostic measures to exclude potential infectious causes should be conducted after CNS events ≥ CTCAE grade 3; an assessment of cerebrospinal fluid should be performed for cytology, cell count, B- and T- cell measurement (flow cytometry at local lab), and viral studies (HSV 1/2, HSV6, JC virus and adenovirus). Additional investigations of the CSF should be performed as clinically appropriate.

For subjects who experience a CTCAE grade 3 CNS adverse event or serious adverse event leading to treatment interruption, if the event has decreased to at least CTCAE grade 1 within 1 week, treatment may be restarted within 2 weeks, but not earlier than 72 hours (3 days) after the infusion was stopped.

After treatment interruption, a new treatment cycle may be started after consultation with the principal investigator. A contrast-enhanced magnetic resonance imaging (MRI) of the head will be performed for subjects who had to interrupt treatment because of a CNS event grade 3 before treatment is resumed. Infusion should be restarted in the hospital, and the subject should remain hospitalized for at least 2 days. Following dexamethasone premedication as described in **Table A**, a new treatment cycle will start with a dose of 9 μg/day. There will be no dose escalation on day 8 or for the following cycles.

If the CNS event was a seizure (CTCAE grade 2 or above), appropriate prophylactic anticonvulsant treatment will be administered during the next treatment cycle.

A grade 3 CNS event leading to treatment interruption at the dose of 9 μg/day or a CNS event needing more than 1 week to resolve to grade ≤ 1 will result in permanent treatment discontinuation.

In case of CNS-related events CTCAE grade 4, or in case of occurrence of more than one seizure, the infusion of blinatumomab will have to be stopped immediately and treatment will be permanently discontinued.

At the discretion of the investigator, an MRI should be considered for subjects who permanently discontinue treatment because of a CNS event ≥ grade 3.

**Table A.** Dexamethasone premedication

| **Treatment phase** | **Target patient** | **Dexamethasone dose** |
| --- | --- | --- |

| Pre-dose dexamethasone before each blinatumomab treatment | All patients (before each cycle and dose increase) | Dexamethasone 20 mg IV within 1 hour before start of treatment in each  treatment cycle, and within 1 hour before dose increase |
| --- | --- | --- |
| Infusion interruption/dose modification due to adverse event | Patients who interrupt treatment > 4 hours | Dexamethasone 20 mg IV within 1 hour before re-start of treatment |
| In case of signs of cytokine release (CRS) | Patients with signs of CRS | Dexamethasone orally or IV at a dose 8 mg every 8 hours for up to 3 days. The dose should then be reduced step-wise over 4 days |
| Infusion interruption/dose modification due to CNS events | Patients with CNS- related AE | Dexamethasone should be administered at a dose of at least 24mg/day. Dexamethasone will then be  reduced step-wise over 4 days. |

- 1. **Ponatinib**
- Ponatinib 15mg tablets will be provided by Takeda and be dispensed from the MDACC outpatient pharmacy. During cycle 1 it will be given as 30mg PO daily continuously (may shift ± 4-5 days if therapy changed once results of cytogenetics known).
- For subsequent courses ponatinib will be administered as 30mg PO daily continuously without interruptions unless due to toxicity. Beginning in cycle 2, in patients who achieved complete molecular response (CMR), ponatinib should be further reduced to 15 mg per day unless judged necessary to stay on 30 mg and after discussion with the PI.
- Ponatinib may be taken with or without food. Patients should avoid grapefruit products while taking ponatinib.
- Avoid coadministration of ponatinib with strong CYP3A inhibitors. If coadministration of a strong CYP3A inhibitor cannot be avoided, reduce the dosage of ponatinib as recommended below.
- After the strong CYP3A inhibitor has been discontinued for 3 to 5 elimination half- lives, resume the ponatinib dosage that was tolerated prior to initiating the strong CYP3A inhibitor.
- Variations in dose adjustment times will not result in protocol deviation.

**Recommended Ponatinib Dosage for Coadministration of Strong CYP3A Inhibitors**

| **Current Ponatinib Dosage** | **Recommended Ponatinib Dosage with a Strong CYP3A Inhibitor** |
| --- | --- |
| 30 mg orally once daily | 15 mg orally once daily |
| 15 mg orally once daily | 10 mg orally once daily |
| 10 mg orally once daily | Avoid coadministration of Ponatinib with a strong CYP3A inhibitor |

- - 1. **Ponatinib Toxicity Management**
- Grade Ill-IV non-hematological toxicity:
  - Consider holding until grade I or less.
  - May consider resuming at 30 mg po daily for grade Ill or IV non-hematological toxicity; consider discontinuing if grade III or IV non-hematological toxicity recurs. The dose can be further decreased to 15 mg daily if needed for serious toxicities, or if found to be in the best interest of the patient and after discussion with the Principal investigator.
- Grade II transaminase elevation
  - Consider holding until grade I or less.
  - Can resume at 30 mg po daily after resolution; reduce dose to 15mg po daily if toxicity recurs.
- Other liver function abnormalities

For patients who develop AST or ALT of ≥ 3x ULN and a total bilirubin of >2 x ULN with alkaline phosphatase of <2 x ULN while on treatment, ponatinib should be held until abnormalities are grade I or less, at which time re-initiation with dose reduction is recommended. Investigation of other causes of liver abnormalities (e.g. azoles) is recommended. Discontinuation of ponatinib is recommended if these abnormalities are deemed by the treating physician to be due to ponatinib. Elevation of AST or ALT

≥ 3 x ULN concurrent with an elevation of bilirubin > 2 x ULN and alkaline phosphatase < 2 x ULN, physician should consider discontinuation.

- Pancreatitis
  - If asymptomatic grade 1-2, considering holding ponatinib until resolution then resume at same dose
  - If asymptomatic or symptomatic grade 3, hold ponatinib until grade 1 and symptoms resolved (if symptomatic), then consider dose reduction (to 30mg or 15mg at discretion of physician). For patients already receiving 15mg of ponatinib, discontinuation is recommended, although resuming again at 15mg can be considered by the treating physician and after discussion with the PI if deemed to be in the best interest of the patient.
  - If grade 4, discontinue ponatinib
- Arterial or venous thrombosis
  - For patients with arterial thrombosis, discontinuation of ponatinib is recommended
  - For patients with pulmonary embolism or lower extremity deep venous thrombosis, dose reduction or discontinuation of ponatinib is recommended.
  - For patients with superficial venous thrombosis or line-associated upper extremity deep venous thrombosis, ponatinib may be continued at the same dose, although holding dose and/or dose reduction can be considered at the discretion of the treating physician according to the assessment of the patient’s best interest. Discontinuation is not recommended in these circumstances.
  - There are no specific recommendations for deep venous thrombosis prophylaxis, although this may be given at the discretion of the treating physician, based on the patient’s risk for thrombosis and bleeding
  - Treatment of arterial and venous thrombosis will be individualized based on the type of thrombosis and the patient’s risk of bleeding
- Heart failure
  - If grade 1-2, considering holding ponatinib until resolution then resume at same dose
  - If grade 3, hold ponatinib until grade 1 or resolved, then consider dose reduction (to 30mg or 15mg at discretion of physician)
  - If grade 4, discontinue ponatinib
- Other modifications should be discussed with the Principal Investigator
  1. **Maintenance Ponatinib**
- Maintenance therapy will consist of single agent ponatinib. Patient with loss of molecular response (PCR>0.01%) can be re-challenged with the blinatumomab up to 4 cycles. Blinatumomab will be administered as continuous intravenous infusion at a dose of 28 μg/24 hours over 4 weeks followed by a treatment-free period of 2 weeks, defined as one 6-week treatment cycle for up to 4 cycles. The Blinatumomab will be performed at MD Anderson Cancer Center. Therapy will be continued for at least 5 years and may continue indefinitely until patients develop resistance/progression or develop an unacceptable toxicity. Management of toxicity is as in section 4.4

1. **CONCOMITANT MEDICATIONS**

Short-acting antacid agents may be taken, but it is recommended that these not be taken from 2 hours before to 2 hours after dosing of ponatinib. Proton pump inhibitors may be administered concomitantly with ponatinib if felt to be in the patient’s best interest.

Corticosteroid use should be limited or avoided. Dexamethasone for the prevention and treatment of Cytokine Release Syndrome (CRS) will be permitted. Any steroid use for anti-leukemic intent will not be permitted.

Patients currently taking drugs that are generally accepted to have a risk of causing Torsades de Pointes (including: quinidine, procainamide, disopyramide, amiodarone, sotalol, ibutilide, dofetilide, erythromycins, clarithromycin, chlorpromazine, haloperidol, mesoridazine, thioridazine, pimozide, cisapride, bepridil, droperidol, methadone, arsenic, chloroquine, domperidone,

halofantrine, levomethadyl, pentamidine, sparfloxacin, lidoflazine) should change these to acceptable alternatives.

Medications that inhibit platelet function (i.e.,aspirin, dipyridamole, epoprostenol, eptifibatide, clopidogrel, cilostazol, abciximab, ticlopidine, and any non-steroidal anti-inflammatory drug) or Anticoagulants (warfarin, heparin/low molecular weight heparin [e.g., danaparoid, dalteparin, tinzaparin, enoxaparin]) should be avoided as much as possible during induction and consolidation due to expected thrombocytopenia.

Exceptions include heparin-flushes for IV lines. If patients develop venous thromboembolism during the course of therapy or are receiving anticoagulation for indications such as recent thrombosis or artificial heart valves these drugs may be continued with close monitoring of the patients.

When used concomitantly with drugs known to be CYP3A4 inducers such as rifampin, consider increasing the dose of ponatinib with careful monitoring for toxicity. When used concomitantly with strong CYP3A4 inhibitors, such as ketoconazole, consider decreasing the ponatinib dose. Dose adjustments for CYP inhibitors/inducers should be conducted after discussion with the principal investigator, and the specific dose increase or decrease will be determined based on the specific drug interaction after discussion with the principal investigator.

Patients may be concurrently enrolling in supportive care clinical trials. Other investigational agents that are used for treatment of other cancers will not be allowed.

1. **STUDY PROCEDURES**
   1. **PRETREATMENT EVALUATION** – within 28 days of prior treatment dosing
      1. Sign Informed Consent
      2. History and physical examination and vital signs, documentation of disease.
      3. Concomitant medication and assessment
      4. Performance status assessment
      5. CBC, platelet count, differential, total bilirubin, creatinine, SGPT or SGOT, serum lipase, serum amylase, triglycerides, uric acid, troponin T.
      6. Bone marrow aspirate and cytogenetics. Quick Philadelphia screen is acceptable. QT-PCR for BCR-ABL. Standard of care bone marrow aspiration and cytogenetics can be used as pretreatment evaluation as long as performed within 28 days prior treatment dosing.
      7. EKG
      8. Urine or serum pregnancy test in patients of appropriate age and menopausal state
      9. Echocardiogram or MUGA scan to assess cardiac function
      10. A chest radiograph should be performed at baseline
      11. Peripheral blood for correlative studies of 10 mL to Dr. Kornblau’s lab for storage.

*Note:*

Standard of care EKG, echocardiogram or MUGA scan, chest radiograph can be used as pretreatment evaluation as long as performed within 28 days prior treatment dosing.

- 1. **EVALUATION DURING STUDY**
     1. 1-2 times weekly CBC, platelet count and differential for course 1 (if in CR at study entry every 1-3 weeks), then every 1-3 weeks during 4 cycles of immunotherapy (C2 – C5) and then every 4-8 weeks during maintenance up to 3 years from C1D1 and then every 3-6 months.
     2. Total bilirubin, SGPT or SGOT, and creatinine weekly for course 1, then every 1-3 weeks during intensive immunotherapy cycles (C2 – C5), then every 4-8 weeks during maintenance up to 3 years from C1D1 and then every 3-6 months. Serum lipase, serum amylase, triglycerides and uric acid as clinically indicated.
     3. Troponin T will be drawn anytime during Cycle 2.
     4. Peripheral PCR will be done every week for the first 4 weeks during Cycle 1 and then every 3-6 months during maintenance after 3 years from C1D1.
     5. Bone marrow aspiration for course 1 (in patients enrolled with active marrow disease) on day 28 (± 7 days) and day 42 (±7 days) (or at the time of hematologic recovery) if day 28 bone marrow with residual leukemia. In CR, marrow every 2 courses (end of C2 and C4 for patients with WBC< 75K/uL, and end of C6 for patients with WBC≥75K/uL and patients with CNS disease at baseline) during consolidation, then every 3-6 months during maintenance up to 3 years from C1D1 and then every year as indicated. Patients not in CR by the end of cycle 1, should also have a bone marrow aspiration performed at the end of cycle 2. When possible, add cytogenetics, quantitative PCR, and mutations.
     6. With any bone marrow aspiration procedure during the first 5 cycles of treatment and at the time of relapse, additional correlative studies maybe performed including genomic testing, methylation profile, next generation sequencing minimal residual disease (NGS MRD) assays using clonoseq by adaptive and correlative studies evaluation of immune cell subsets (5-10mL of bone marrow aspirate to Dr. Kornblau’s lab).
     7. Evaluate for toxicity assessment starts from first dosing day (C1D1) until 30 days after last dose of study drug/s.
     8. EKG – Recommended at week 4
     9. At each study visit concomitant medication and assessment will be recorded.
     10. ECHO or MUGA as clinically indicated
     11. Peripheral blood for correlative studies for germline control will be collected within 60 days from first documentation of CR/CRp/CRi and stored. Peripheral blood 10mL to Dr. Kornblau’s lab.
     12. Peripheral blood for NGS MRD analysis correlative studies. Course 1, day 8 (± 3 days), day 15 (± 3 days), day 22 (± 3 days) and day 29 (± 3 days).
     13. Each time lumbar puncture is performed during the study, sample will be sent to flow cytometry for MRD as standard of care procedure.
     14. For the surveillance/ monitoring lumbar puncture after completion of the combination, sample will be sent for chemistry, hematology, cytology and MRD by flow cytometry as standard of care every 3 months (± 1 month) for 2 years from the completion date

Correlative studies may be omitted on an individual basis after discusson with the principal investigator. Correlative samples not obtained related to logistical reasons will not be considered a deviation. Missed correlative studies will not be considered a deviation.

Lumbar punctures that are not obtained related to logistical reasons will not be considered a deviation. Missed lumbar puncture samples will not be considered a deviation.

|  | **Patients with WBC<75K/uL: Blinatumomab+Ponatinib (C1-5), and patients with**  **WBC≥75K/uL and patients with CNS disease at baseline: Blinatumomab+ponatinib (C1-2 and C5-6) and MTX+cytarabine+blinatumomab+ponatinib (C3-4)** | | | | | | | | **Ponatinib** | **Follow-up** |
| --- | --- | --- | --- | --- | --- | --- | --- | --- | --- | --- |
|  | | **Pre-** | **Cycles** | | | | | |  |  |
|  |  |  | **1** | **2** | **3** | **4** | **5** | **6** | **Maintenance** | **30 Days after last dose of study drugs and every 6 months**  **thereafter (may be by phone call)6** |
| Informed consent | | X |  |  |  |  |  |  |  |  |
| EKG | | X | Week 4 |  |  |  |  |  |  |  |
| History + physical + documentation of  disease | | X |  |  |  |  |  |  |  |  |
| Vital signs | | X |  |  |  |  |  |  |  |  |
| Performance Status | | X |  |  |  |  |  |  |  |  |
| CBC/Diff/Platelet1 | | X | 1-2 times weekly | Every 1-3 weeks | | | | | Every 4-8 weeks up to 3 years from C1D1 and then  every 3-6 months |  |
| Total bilirubin, creatinine, SGPT or SGOT, serum lipase, serum amylase, triglycerides, uric acid.  10 | | X | Weekly9 | Every 1-3 weeks9 | | | | | Every 4-8 weeks up to 3 years from C1D1 and then every 3-6 months 9 |  |

| Marrow2  Cytogenetics2 MRD | X3 | D28, D424 | X |  |  | X | X | Every 3-6 months during maintenance up to 3 years from C1D1 and then every year as  clinically indicated |  |
| --- | --- | --- | --- | --- | --- | --- | --- | --- | --- |
| Peripheral PCR |  | X13 |  |  |  |  |  | every 3-6 months during maintenance after 3 years from C1D1 |  |
| Troponin T | X |  | X12 |  |  |  |  |  |  |
| Urine or serum pregnancy test (If  indicated) | X |  |  |  |  |  |  |  |  |
| Echocardiogram or MUGA5 | X |  |  |  |  |  |  |  |  |
| Chest X-Ray | X |  |  |  |  |  |  |  |  |
| Peripheral blood T-cell correlative studies7 | X | D7, D28 |  |  |  |  |  |  |  |
| Peripheral blood NGS MRD correlative studies8 |  | D8, D15, D22, D29 |  |  |  |  |  |  |  |
| Peripheral correlative studies for germline control9 |  |  | Collected within 60 days  from the first documentation of CR/CRp/CRi | | | | |  |  |
| Concomitant medication and assessment | X |  | At every visit (every 1-3 courses) | | | | | Every 3-6 months |  |
| Toxicity assessment11 |  | X | X | X | X | X | X | X | X |
| Lumbar puncture14, 15 |  | X | X | X | X | X | X | X | Surveillance/ monitoring lumbar puncture after off- treatment as standard of care every 3 months (± 1 month) for 2 years from the off-treatment date |

All patients will also have a bone marrow aspiration performed at the time of relapse to document loss of response. Correlative studies will also be sent on the relapse bone marrow specimen.

1 CBC, platelet count and differential if in CR at study entry every 1-3 weeks, then every 1-3 weeks during 4 cycles of immunotherapy (C2 – C5) and then every 4-8 weeks during maintenance up to 3 years from C1D1 and then every 3-6 months

2 When possible, add cytogenetics mutations, and correlative studies. Quantitative PCR for BCR- ABL should be performed on all bone marrow examinations.

3 Quick Philadelphia screen is acceptable.

4 Bone marrow aspiration Course 1, Day 42 (± 7 days), if Day 28 bone marrow with residual leukemia.

5 As clinically indicated during treatment

6 The phone call should last about 10 minutes.

7 Peripheral blood for correlative studies at screening, Course 1, day 7 (± 3 days) and day 28 (± 3 days).

8 Peripheral blood for NGS MRD analysis. Course 1, day 8 (± 3 days), day 15 (± 3 days), day 22 (±

3 days) and day 29 (± 3 days).

9 Peripheral blood for correlative studies for germline control will be collected within 60 days from first documentation of CR/CRp/CRi and stored. Peripheral blood 10mL to Dr. Kornblau’s lab.

10 Serum lipase, serum amylase, triglycerides, and uric acid as indicated

11 Toxicity assessment starts from first dosing day (C1D1) until 30 days after last dose of study drug/s.

12 Troponin T will be drawn anytime during Cycle 2.

13 Peripheral PCR will be done every week for the first 4 weeks during Cycle 1 and then every 3-6 months during maintenance after 3 years from C1D1.

14 Each time lumbar puncture is performed during the study, sample will be sent to flow cytometry for MRD as standard of care procedure.

15 For the surveillance/ monitoring lumbar puncture after completion of the combination, sample will be sent for chemistry, hematology, cytology and MRD by flow cytometry as standard of care every 3 months (± 1 month) for 2 years from the completion date.

* At least one marrow assessment should be performed at 3 months from the start of treatment (± 2 weeks) for minimal residual disease determination

- 1. **Follow-up**

Thirty days (± 7 days) after last dose of the study drugs and AE assessment will be recorded. This may be done over the phone with a member of the study staff. The phone call should last about 10 minutes.

- 1. **Long-term Follow-up**

Patients will be followed periodically every 6 months (+/- 3 months) for survival via brief phone call, even after being taken off treatment. This may be done over the phone with a member of the study staff. The phone call should last about 10 minutes. For patients registered on our long-term follow-up umbrella protocol DR09-0223, survival follow-up may be conducted under that protocol.

1. **EFFICACY AND SAFETY ASSESSMENTS**
   1. **CRITERIA FOR RESPONSE**
      1. Complete Remission (CR): Normalization of the peripheral blood and bone marrow with ≤ 5% blasts in normocellular or hypercellular marrow with a granulocyte count of 1 x 109/L or above, and platelet count of 100 x 109/L. Complete resolution of all sites of extramedullary disease is required for CR.
      2. Complete remission without recovery of counts (CRi): Peripheral blood and marrow results as for CR, but with incomplete recover of counts (platelets < 100 x 109/L; neutrophils < 1 x 109/L).
      3. Complete cytogenetic response (CCyR): Same as for CR with cytogenetics showing diploid karyotype
      4. Complete molecular response (CMR): Same as for CR with RT-PCR negativity for BCR-ABL or with RT-PCR for BCR-ABL with 4 log reduction from baseline and/or

≤0.01%.

- - 1. Major molecular response (MMR): Same as for CR with RT-PCR for BCR-ABL with 3 log reduction from baseline and/or ≤0.1% by the International Scale
    2. Partial Response (PR): As above for CR except for the presence of 6-25% marrow blasts.
    3. Relapse and resistant disease will be defined based on morphological assessment of bone marrow and peripheral blood.
    4. Minimal Residual Disease (MRD): MRD levels will be continuously assessed during induction and consolidation therapy by 6-color multiparameter flow. MRD negativity will be defined by a value of at least 10−4 and confirmed on a second bone marrow aspiration/biopsy performed after a subsequent cycle. In addition, patients should have normalization of the peripheral blood and bone marrow with 5% or less blasts in a normocellular or hypercellular marrow with a granulocyte count of 1 x 109/L or above and a platelet count of 100 x 109/L or above.
  1. **EVALUATION OF TOXICITY**
     1. Toxicities will be graded according to the NCI Common Toxicity Criteria for Adverse Event Reporting Version 4.0. The toxicity of the regimen will be monitored continuously during the course of the study.
     2. Ponatinib will be placed on hold in case of worsening of heart failure, hepatotoxicity, venous/arterial thromboembolism, pancreatitis, or CTCAE v4.0 grade III/IV toxicity.
  2. **CRITERIA FOR REMOVAL FROM THE STUDY**
     1. Unacceptable toxicity judged to be related to therapy by the investigator, as defined by the NCI Common Toxicity Criteria (Irreversible or prolonged (greater than 42 days) grade 4 hematological toxicity or grade 4 non-hematological toxicity thought to be related to ponatinib).
     2. Non-compliance by the patient with protocol requirements or patient's request to be removed from the study.
     3. Disease progression.

Any expired or unused drug study medications will be destroyed according to institutional policy. This will include also any leftover medication returned by the patient.

- 1. **DEFINITION OF STUDY END-POINTS**
     1. **Relapse-free survival** is the time from documented complete response until relapse or death.
     2. **Event-free survival** is the time from the first day of treatment until any failure (resistant disease, relapse, or death).
     3. **Overall response rate** is defined as the percentage of patients achieving CR or CRi
     4. **Overall survival** is defined as the time from the first day of treatment to time of death from any cause.

1. **REPORTING REQUIREMENTS**

**Pregnancy**

It is not known what effects ponatinib has on human pregnancy or development of the embryo or fetus. Therefore, female patients participating in this study should avoid becoming pregnant, and male patients should avoid impregnating a female partner. Nonsterilized female patients of reproductive age group and male patients should use effective methods of contraception through defined periods during and after study treatment as specified below.

Female patients must meet 1 of the following:

- Postmenopausal for at least 1 year before the screening visit, or
- Surgically sterile, or
- If they are of childbearing potential, agree to practice 2 effective methods of contraception from the time of signing of the informed consent form through 4 months after the last dose of study drug, or agree to completely abstain from heterosexual intercourse.

Male patients, even if surgically sterilized (i.e., status post-vasectomy) must agree to 1 of the following:

- Practice effective barrier contraception during the entire study treatment period and through 4 months after the last dose of study drug, or completely abstain from heterosexual intercourse.

1. **ADVERSE EVENTS**
   1. **Definitions**
      1. **Adverse Event Definition**

Adverse event (AE) means any untoward medical occurrence in a patient or subject administered a medicinal product; the untoward medical occurrence does not necessarily have a causal relationship with this treatment. An AE can therefore be any unfavorable and unintended sign (including an abnormal laboratory finding), symptom, or disease temporally associated with the use of a medicinal (investigational) product whether or not it is related to the medicinal product. This includes any newly occurring event, or a previous condition that has increased in severity or frequency since the administration of study drug.

An abnormal laboratory value, vital sign or other diagnostic test result will not constitute an AE unless that value leads to discontinuation or delay in treatment, dose modification or therapeutic intervention.

- - 1. **Adverse Drug Reaction**

An adverse drug reaction (ADR) is a response to a medicinal product which is noxious and unintended. Response in this context means that a causal relationship between a medicinal product and an adverse event is at least a reasonable possibility. This includes adverse reactions which arise from: use of a medicinal product within the terms of the marketing authorization; use outside the terms of the marketing authorization, including overdose, misuse, abuse and medication errors; and occupational exposure*.

* This corresponds to the exposure to a medicinal product for human use as a result of one’s occupation, such as nurses who may handle products routinely in their occupational setting.

- - 1. **Serious Adverse Event Definition**

Serious AE (SAE) means any untoward medical occurrence that at any dose:

- - - - Results in **death.**
      - Is **life-threatening** (refers to an AE in which the patient was at risk of death at the time of the event. It does not refer to an event which hypothetically might have caused death if it were more severe).
      - Requires inpatient **hospitalization or prolongation of an existing hospitalization**

(see clarification in the paragraph below on planned hospitalizations).

- - - - Results in **persistent or significant disability or incapacity**. (Disability is defined as a substantial disruption of a person’s ability to conduct normal life functions).
      - Is a **congenital anomaly/birth defect**.
      - Is a **medically important event**. This refers to an AE that may not result in death, be immediately life threatening, or require hospitalization, but may be considered serious when, based on appropriate medical judgment, may jeopardize the patient, require medical or surgical intervention to prevent 1 of the outcomes listed above, or involves suspected transmission via a medicinal product of an infectious agent. Examples of such medical events include allergic bronchospasm requiring intensive treatment in an emergency room or at home, blood dyscrasias or convulsions that do not result in inpatient hospitalization, or the development of drug dependency or drug abuse; any organism, virus, or infectious particle (e.g., prion protein transmitting Transmissible Spongiform Encephalopathy), pathogenic or nonpathogenic, is considered an infectious agent.

Clarification should be made between a serious AE (SAE) and an AE that is considered severe in intensity (Grade 3 or 4), because the terms serious and severe are NOT synonymous. The general term *severe* is often used to describe the intensity (severity) of a specific event; the event itself, however, may be of relatively minor medical significance (such as a Grade 3 headache). This is NOT the same as *serious*, which is based on patient/event outcome or action criteria described above, and is usually associated with events that pose a threat to a patient’s life or ability to function. A severe AE (Grade 3 or 4) does not necessarily need to be considered serious. For example, a white blood cell count of 1000/mm3 to less than 2000 is considered Grade 3 (severe) but may not be considered serious. Seriousness (not intensity) serves as a guide for defining regulatory reporting obligations.

- 1. **Procedures for Recording and Reporting Adverse Events**

Adverse Events may be spontaneously identified by the patient and/or in response to an open question from study personnel or revealed by observation, physical examination, or other diagnostic procedures. Any clinically relevant deterioration in laboratory assessments or other clinical finding is considered an AE. When possible, signs and symptoms indicating a common underlying pathology should be noted as one comprehensive event.

An adverse event is the appearance or worsening of any undesirable sign, symptom, or medical condition occurring after starting the study drug even if the event is not considered to be related to study drug. Medical conditions/diseases present before starting study drug are only considered adverse events if they worsen after starting study drug.

The investigator (or physician designee) is responsible for verifying and providing source documentation for all adverse events and assigning the attribution for all adverse events for subjects enrolled. The Principal Investigator will sign and date the Adverse Event Record (AE logs) per patient after the completion of each course. Following signature, the AE logs will be used as source documentation for the adverse events for attribution.

Adverse event reporting will be as per the NCI criteria and the MDACC Leukemia Specific Adverse Event Recording and Reporting Guidelines (Appendix C).

The descriptions and grading scales found in the revised NCI Common Terminology Criteria for Adverse Events (CTCAE) version 4.0 will be utilized for adverse event reporting. ([http://ctep.cancer.gov/reporting/ctc.html).](http://ctep.cancer.gov/reporting/ctc.html))

**Serious Adverse Event Reporting (SAE) for M. D. Anderson-sponsored IND Protocols**

An adverse event or suspected adverse reaction is considered “serious” if, in the view of either the investigator or the sponsor, it results in any of the following outcomes:

- Death
- A life-threatening adverse drug experience – any adverse experience that places the patient, in the view of the initial reporter, at immediate risk of death from the adverse experience as it occurred. It does not include an adverse experience that, had it occurred in a more severe form, might have caused death.
- Inpatient hospitalization or prolongation of existing hospitalization
- A persistent or significant incapacity or substantial disruption of the ability to conduct normal life functions.
- A congenital anomaly/birth defect.

Important medical events that may not result in death, be life-threatening, or require hospitalization may be considered a serious adverse drug experience when, based upon appropriate medical judgment, they may jeopardize the patient or subject and may require medical or surgical intervention to prevent one of the outcomes listed in this definition. Examples of such medical events include allergic bronchospasm requiring intensive treatment in an emergency room or at home, blood dyscrasias or convulsions that do not result in inpatient hospitalization, or the development of drug dependency or drug abuse (21 CFR 312.32).

- Important medical events as defined above, may also be considered serious adverse events. Any important medical event can and should be reported as an SAE if deemed appropriate by the Principal Investigator or the IND Sponsor, IND Office.
- All events occurring during the conduct of a protocol and meeting the definition of a SAE must be reported to the IRB in accordance with the timeframes and procedures outlined in “The University of Texas M. D. Anderson Cancer Center Institutional Review Board Policy for Investigators on Reporting Unanticipated Adverse Events for Drugs and Devices”. Unless stated otherwise in the protocol, all SAEs, expected or

unexpected, must be reported to the IND Office, regardless of attribution (within 5 working days of knowledge of the event).

- **All life-threatening or fatal events,** that are unexpected, and related to the study drug, must have a written report submitted within **24 hours** (next working day) of knowledge of the event to the Safety Project Manager in the IND Office.
- Unless otherwise noted, the electronic SAE application (eSAE) will be utilized for safety reporting to the IND Office and MDACC IRB.
- Serious adverse events will be captured from the time of the first protocol-specific intervention, until 30 days after the last dose of drug, unless the participant withdraws consent. Serious adverse events must be followed until clinical recovery is complete and laboratory tests have returned to baseline, progression of the event has stabilized, or there has been acceptable resolution of the event.
- Additionally, any serious adverse events that occur after the 30 day time period that are related to the study treatment must be reported to the IND Office. This may include the development of a secondary malignancy.

**Reporting to FDA:**

Serious adverse events will be forwarded to FDA by the IND Sponsor (Safety Project Manager IND Office) according to 21 CFR 312.32.

It is the responsibility of the PI and the research team to ensure serious adverse events are reported according to the Code of Federal Regulations, Good Clinical Practices, the protocol guidelines, the sponsor’s guidelines, and Institutional Review Board policy.

**Investigator Communication with Supporting Companies:**

Adverse Events which are **serious** must be reported to Takeda Pharmacovigilance (or designee) from the first dose of ponatinib up to and including 30 days after administration of the last dose of ponatinib. Any SAE that occurs at any time after completion of ponatinib treatment or after the designated follow-up period that the sponsor-investigator and/or sub- investigator considers to be related to any study drug must be reported to Takeda Pharmacovigilance (or designee). In addition, new primary malignancies that occur during the follow-up periods must be reported, regardless of causality to study regimen, for a minimum of three years after the last dose of the investigational product, starting from the first dose of study drug. All new cases of primary malignancy must be reported to Takeda Pharmacovigilance (or designee).

Planned hospital admissions or surgical procedures for an illness or disease that existed before the patient was enrolled in the trial are not to be considered AEs unless the condition

deteriorated in an unexpected manner during the trial (e.g., surgery was performed earlier or later than planned). All SAEs should be monitored until they are resolved or are clearly determined to be due to a patient’s stable or chronic condition or intercurrent illness(es).

The principal investigator, Dr. Elias Jabbour, also referred to as the sponsor-investigator, is responsible for reporting serious adverse events (SAEs) to any regulatory agency and to the sponsor- investigator’s EC or IRB.

Regardless of expectedness or causality, all SAEs must also be reported in English to Takeda Pharmacovigilance or designee:

**Fatal and Life Threatening SAEs** within 24 hours of the -investigator’s observation or awareness of the event

**All other serious (non-fatal/non-life threatening) events** within 4 calendar days of the investigator’s observation or awareness of the event

These serious events must be reported to the company within the timelines specified for immediate reporting. The SAE report must include at minimum:

- **Event term(s)**
- **Serious criteria**
- **Intensity of the event(s):** Sponsor-investigator’s or sub-investigator’s determination. Intensity for each SAE, including any lab abnormalities, will be determined by using the NCI CTCAE version specified in the protocol, as a guideline, whenever possible. The criteria are available online at [http://ctep.cancer.gov/reporting/ctc.html.](http://ctep.cancer.gov/reporting/ctc.html)
- **Causality of the event(s):** Sponsor-investigator’s or sub-investigator’s determination of the relationship of the event(s) to study drug administration.

Follow-up information on the SAE may be requested by Takeda Pharmacovigilance (or designee).

In the event that this is a multisite study, the sponsor-investigator is responsible to ensure that the SAE reports are sent to Takeda Pharmacovigilance (or designee) from all sites participating in the study. Sub-investigators must report all SAEs to the sponsor- investigator so that the sponsor-investigator can meet his/her foregoing reporting obligations to the required regulatory agencies and to Takeda Pharmacovigilance, unless otherwise agreed between the sponsor-investigator and sub-investigator(s).

Relationship to all study drugs for each SAE will be determined by the investigator or sub- investigator by responding yes or no to the question: Is there a reasonable possibility that the AE is associated with the study drug(s)?

Adverse Events of Special Interest (AESIs)

Vascular occlusive events have been identified as AESIs for ponatinib. These include arterial and venous thrombotic and occlusive adverse events that meet the criteria for SAEs (cross-refer to the section where the serious criteria are described and defined) and those adverse events that do not meet the SAE criteria. AESIs require ongoing monitoring by investigators and rapid identification and communication by the investigator to the supporting company. All AESIs, whether SAEs or not, must be reported within 2 business days of the Principal Investigator (PI) awareness to Takeda. Takeda has determined that the events listed below (whether considered serious or non-serious by investigators) should be considered AESIs:

1. Myocardial infarction: The Third Universal Definition of Myocardial Infarction (Thygesen et al, 2012) is used to define MI
2. Angina (newly diagnosed or worsening of existing angina or unstable angina)
3. Coronary artery disease (CAD) (newly diagnosed or worsening of existing CAD) or symptoms that may reflect cardiovascular disease (Thygesen et al, 2012)
4. Cerebrovascular ischemic disease including ischemic or hemorrhagic stroke, vascular stenosis, transient ischemic accident (TIA), cerebrovascular occlusive disease documented on diagnostic neuroimaging, or symptoms that may reflect cerebrovascular disease (Easton et al, 2009)
5. New onset or worsening of peripheral artery occlusive disease (eg, renal artery, mesenteric artery, femoral artery) or symptoms that may reflect peripheral vascular disease
6. Retinal vascular thrombosis, both venous and arterial
7. Venous thromboembolism where significant compromise of organ function or other significant consequences could result (eg, pulmonary embolism, portal vein thrombosis, renal vein thrombosis) or symptoms that may reflect venous thrombosis

TAKEDA may request additional information from the PI on observed AESIs and this information should be provided in a timely fashion (ie, within 2 business days of the PI awareness).

All serious adverse events, whether “reportable” as defined in this protocol or not, must be reported to TAKEDA. All expedited (7/15 day) reports will be sent to TAKEDA simultaneously or within 24 hours of PI’s submission to the competent authorities. Non- expedited SAE reports (except for AESIs) can be batched by the PI and sent to TAKEDA on a monthly basis. Also, any event of a vascular occlusive nature, either serious or non- serious, must be reported to TAKEDA **within 4 calendar days** of the PI’s awareness.

The study PI or designee is responsible for faxing SAE reports to TAKEDA, at 1-800-963- 6290or email: [takedaoncocases@cognizant.com](mailto:takedaoncocases@cognizant.com)

**References quoted in the above text:**

Thygesen K, Alpert JS, Jaffe AS, et al. Third Universal Definition of Myocardial Infarction. Circulation. 2012;126 (16):2020-2035. Version 2.0_01 Apr 2015

Easton JD, Saver JL, Albers GW, et al. Definition and evaluation of transient ischemic attack: A Scientific Statement for Healthcare Professionals From the American Heart Association/American Stroke Association Stroke Council; Council on Cardiovascular Surgery and Anesthesia; Council on Cardiovascular Radiology and Intervention; Council on Cardiovascular Nursing; and the Interdisciplinary Council on Peripheral Vascular Disease. Stroke. 2009;40 (6):2276-2293.

**Investigator Communication with Amgen:**

All suspected unexpected serious adverse reactions (SUSARs) related or possibly related to blinatumomab and their follow-up reports must be reported to Amgen within 24 hours of submission to the regulatory agency, IRB or IEC. A copy of any safety report involving an Amgen drug (e.g. blinatumomab) submitted to the regulatory agency, IRB or IEC, must be faxed to Amgen, within 24 hours of such submission.

The Investigator must report all pregnancies and pregnancies occurring in the partner of a patient participating in the study or potential infant exposure through lactation within 10 calendar days of sponsor’s awareness to Amgen.

SAE will be captured in SAE log and submitted to Amgen every 6 months via fax or email.

Fax: 888-814-8653 (toll-free, US) 805-480-9205 (toll)

E-mail: [svc-ags-in-us@amgen.com](mailto:svc-ags-in-us@amgen.com)

- 1. **Procedures for Reporting Drug Exposure During Pregnancy and Birth Events**

If a woman becomes pregnant or suspects that she is pregnant while participating in this study, she must inform the investigator immediately and permanently discontinue study drug. The sponsor-investigator must fax a completed Pregnancy Form to the Takeda Pharmacovigilance or designee immediately*.* The pregnancy must be followed for the final pregnancy outcome (i.e., delivery, still birth, miscarriage) and Takeda Pharmacovigilance or designee will request this information from the sponsor-investigator.

If a female partner of a male patient becomes pregnant during the male patient’s participation in this study, the sponsor-investigator must also immediately fax a completed Pregnancy Form to the Takeda Pharmacovigilance or designee. Every effort should be made to follow the pregnancy for the final pregnancy outcome.

1. **ADMINISTRATIVE REQUIREMENTS**
   1. **Product Complaints or Medication Errors (Including Overdose)**

A product complaint is a verbal, written, or electronic expression that implies dissatisfaction regarding the identity, strength, purity, quality, or stability of a drug product. Individuals who identify a potential product complaint situation should immediately contact Takeda Pharmacovigilance or designee (see below) and report the event. Whenever possible, the

associated product should be maintained in accordance with the label instructions pending further guidance from a Takeda Quality representative.

A medication error is a preventable event that involves an identifiable patient and that leads to inappropriate medication use, which may result in patient harm. While overdoses and underdoses constitute medication errors, doses missed inadvertently by a patient do not. Investigators must record all medication errors (including overdose) on the appropriate CRF form. Individuals who identify a potential medication error situation should immediately contact Takeda (see below) and report the event.

**For Product Complaints or Medication Errors (Including Overdose), contact Takeda Pharmacovigilance**

**E-mail:** [GlobalOncologyMedinfo@takeda.com](mailto:GlobalOncologyMedinfo@takeda.com)

**Tel:** 1-844-T1-POINT (1-844-817-6468) Hours: Mon-Fri, 9 a.m. – 7 p.m. ET

Product complaints in and of themselves are not AEs. If a product complaint results in an SAE, an SAE form should be completed and sent to Takeda

1. **OUTSIDE PHYSICIAN PARTICIPATION DURING TREATMENT**
   1. MDACC Physician communication with the outside physician is required prior to the patient returning to the local physician. This will be documented in the patient record.
   2. A letter to the local physician outlining the patient's participation in a clinical trial will request local physician agreement to supervise the patient's care
   3. Protocol required evaluations outside MDACC will be documented by telephone, fax or e- mail. Fax and/or e-mail will be dated and signed by the MDACC physician, indicating that they have reviewed it.
   4. Changes in drug dose and/or schedule must be discussed with and approved by the MDACC physician investigator, or their representative prior to initiation, and will be documented in the patient record.
   5. A copy of the informed consent, protocol abstract, treatment schema and evaluation during treatment will be provided to the local physician.
   6. Documentation to be provided by the local physician will include drug administration records, progress notes, reports of protocol required laboratory and diagnostic studies and documentation of any hospitalizations.
   7. The home physician will be requested to report to the MDACC physician investigator all life threatening events within 24 hours of documented occurrence.
   8. Patients will return to MDACC every 1 – 3 courses during consolidation, then every 3-6 months during maintenance for evaluation.
2. **STATISTICAL METHODOLOGY**

A maximum of 120 patients will be enrolled in this Phase II study, which includes 90 newly diagnosed elderly (≥60 years) Ph-positive and/or BCR-ABL-positive patients (cohort 1) and 30 patients with relapsed/refractory disease (cohort 2). The primary objective is to evaluate complete molecular response (Same as for CR [Section 7.1] with RT-PCR negativity for BCR-ABL) in cohort 1 and overall response (CR+CRi) in cohort 2.

For analysis plan of exploratory objectives, please refer to the appendix “Novel Next Generation Sequencing-Based Assays for Risk Assessment in Philadelphia Chromosome-Positive ALL (Ph+ ALL): TAKEDA Correlative Sciences” (Appendix F).

**EFFICACY**

For cohort 1, the target complete molecular response (CMR) rate is 35% and for monitoring purpose, the assessment window for CMR is 3 cycles. This regimen of the combination treatment will be considered worthy of further investigation if it elicits an increase in CMR to 35% with acceptable toxicity. A >30% drug-related grade 3/4 or higher toxicity rate is considered unacceptable. Thus, interim monitoring rules, assuming the prior distributions below, were constructed that meet the following two conditions,

1. Stop if Prob{p(CMR, E)<p(CMR, H) | data} > 0.99, or
2. Stop if Prob{p(TOX, E)>0.20 | data} > 0.85,

where p(CMR, E) and p(TOX, E) are the true CMR and toxicity rates for the combination treatment, and p(CMR, H) is the true CMR rate of the standard treatment. The first rule provides for stopping the study if the data suggest that it is unlikely (i.e., probability < 1%) that CMR rate of the combination treatment is greater than the CMR rate of standard treatment. The second condition will stop the study early if excessive therapy-related grade 3/4 or higher toxicity (>20%) is highly probable (i.e., probability >85%) for the combination treatment. Monitoring for toxicity and futility will not begin until 5 patients have been evaluated, and cohort size for future evaluations is 5.

The monitoring rule for the CMR rate and toxicity rate, based on these assumptions and monitoring conditions above is found in Table 1. For example, accrual will cease if 3 or more patients experience toxicities among the first 5 patients. Accrual will cease if no patients experience a response within 3 cycles in the first 10 patients treated.

| # of patients | Stop the cohort if there are this many CMR | Stop the cohort if there are this many drug-related grade 3 or higher toxicity |
| --- | --- | --- |
| 5 | Never stop | 3 |
| 10 | 0 | 4 |
| 15 | 1 | 5 |
| 20 | 2 | 7 |
| 25 | 3 | 8 |
| 30 | 4 | 9 |
| 35 | 6 | 10 |
| 40 | 7 | 11 |
| 45 | 8 | 13 |
| 50 | 10 | 14 |
| 55 | 11 | 15 |
| 60 | 12 | 16 |
| 65 | 14 | 17 |
| 70 | 15 | 18 |
| 75 | 17 | 19 |
| 80 | 18 | 21 |
| 85 | 19 | 22 |
| 90 | Always stop | Always stop |

Multc Lean Desktop (version 2.1.0) was used to generate the toxicity and futility stopping boundaries and the OC table (Table 2). In order to utilize the software for the design, a 35% constant rate and beta (0.7, 1.3) priors were assumed for the standard treatment response rate and experimental treatment response prior distribution, respectively. In addition, a 20% constant rate and beta (0.4, 1.6) priors were assumed for the standard treatment toxicity rate and experimental treatment toxicity prior distribution, respectively.

The probability of stopping the study early if the true CMR of the combination treatment was 35% and the true toxicity rate was 20% was 41%. Probabilities of stopping early for high true toxicity rates (i.e., 40%) were 99% when the true CMR was 35% and 99% when true CMR rate was 15%.

| Table 2. Operating characteristics for simultaneous monitoring response and toxicity rates for patients treated with combination treatment | | |
| --- | --- | --- |
| True Toxicity Rate | True CMR | Prob(stop the trial early) |
| 0.05 | 0.15 | 0.9876 |
|  | 0.25 | 0.5157 |
|  | 0.35 | 0.0609 |
|  | 0.45 | 0.0069 |

| Table 2. Operating characteristics for simultaneous monitoring response and toxicity rates for patients treated with combination treatment | | |
| --- | --- | --- |
| True Toxicity Rate | True CMR | Prob(stop the trial early) |
|  | 0.55 | 0.0027 |
| 0.10 | 0.15 | 0.9879 |
|  | 0.25 | 0.5271 |
|  | 0.35 | 0.0832 |
|  | 0.45 | 0.0305 |
|  | 0.55 | 0.0263 |
| 0.20 | 0.15 | 0.9922 |
|  | 0.25 | 0.6951 |
|  | 0.35 | 0.4088 |
|  | 0.45 | 0.4748 |
|  | 0.55 | 0.3722 |
| 0.30 | 0.15 | 0.9991 |
|  | 0.25 | 0.9640 |
|  | 0.35 | 0.9303 |
|  | 0.45 | 0.9262 |
|  | 0.55 | 0.9259 |
| 0.40 | 0.15 | 0.9999 |
|  | 0.25 | 0.9997 |
|  | 0.35 | 0.9993 |
|  | 0.45 | 0.9993 |
|  | 0.55 | 0.9993 |

For cohort 2, the target overall response (OR) rate is 50% and for monitoring purpose, the assessment window for OR is 2 cycles. This regimen of the combination treatment will be considered worthy of further investigation if it elicits an increase in OR to 50% with acceptable toxicity. A >30% drug-related grade 3/4 toxicity rate is considered unacceptable. Thus, interim monitoring rules, assuming the prior distributions below, were constructed that meet the following two conditions,

1. Stop if Prob{p(OR, E ) < p(OR, H) | data} > 0.95, or
2. Stop if Prob{p(TOX, E) > 0.30 | data} > 0.85,

where p(OR, E) and p(TOX, E) are the true OR and toxicity rates for the combination treatment, and p(OR, H) is the true OR rate of the standard treatment. The first rule provides for stopping the study if the data suggest that it is unlikely (i.e., probability < 5%) that OR rate of the combination treatment is greater than the OR rate of standard treatment. The second condition will stop the study early if excessive therapy-related grade 3/4 toxicity (>30%) is highly probable (i.e., probability >85%) for the combination

treatment. Monitoring for toxicity and futility will not begin until 2 patients have been evaluated, and cohort size for future evaluations is 2.

The monitoring rule for the toxicity rate, based on these assumptions and monitoring conditions above is found in Table 4. For example, accrual will cease if 3 or more patients experience toxicities among the first 4 patients.

| Table 4. Stop accrual if the number of drug-related grade 3/4 toxicities is greater than or equal to indicated (i.e., # patients with toxicities) among the number of patients evaluated | | | | | | | | | | | | |
| --- | --- | --- | --- | --- | --- | --- | --- | --- | --- | --- | --- | --- |
| # patients evaluated | 2 | 4 | 6 | 8-10 | 12 | 14-16 | 18 | 20 | 22-24 | 26 | 28 | 30 |
| # patients with toxicities | 2 | 3 | 4 | 5 | 6 | 7 | 8 | 9 | 10 | 11 | 12 | Always stop |

Monitoring the OR rate, based on the above assumptions and monitoring conditions is found in Table 5. For example, accrual will cease if no patients experience an overall response within 2 cycles in the first 4 patients treated.

| Table 5. Stop accrual if the number with overall response is less than or equal to indicated (i.e., # patients with overall response) among the number of patients evaluated | | | | | | | | | | | | |
| --- | --- | --- | --- | --- | --- | --- | --- | --- | --- | --- | --- | --- |
| # patients evaluated | 2 | 4-  6 | 8 | 10 | 12-14 | 16 | 18 | 20 | 22-  24 | 26 | 28 | 30 |
| # patients with overall response | Never stop | 0 | 1 | 2 | 3 | 4 | 5 | 6 | 7 | 8 | 9 | Always stop |

Multc Lean Desktop (version 2.1.0) was used to generate the toxicity and futility stopping boundaries and the OC table (Table 6). In order to utilize the software for the design, a 50% constant rate and beta (1.0, 1.0) priors were assumed for the standard treatment response rate and experimental treatment response prior distribution, respectively. In addition, a 30% constant rate and beta (0.6, 1.4) priors were assumed for the standard treatment toxicity rate and experimental treatment toxicity prior distribution, respectively.

The probability of stopping the study early if the true OR of the combination treatment was 50% and the true toxicity rate was 30% was 44%. Probabilities of stopping early for high true toxicity rates (i.e., 50%) were 93% when the true OR was 50% and 98% when true OR rate was 30%.

| Table 6. Operating characteristics for simultaneous monitoring response and toxicity rates for patients treated with combination treatment | | |
| --- | --- | --- |
| True Toxicity Rate | True OR | Prob(stop the trial early) |
| 0.10 | 0.30 | 0.8050 |
|  | 0.40 | 0.4563 |
|  | 0.50 | 0.1773 |
|  | 0.60 | 0.0583 |

| Table 6. Operating characteristics for simultaneous monitoring response and toxicity rates for patients treated with combination treatment | | |
| --- | --- | --- |
| True Toxicity Rate | True OR | Prob(stop the trial early) |
|  | 0.70 | 0.0236 |
| 0.20 | 0.30 | 0.8205 |
|  | 0.40 | 0.4995 |
|  | 0.50 | 0.2427 |
|  | 0.60 | 0.1332 |
|  | 0.70 | 0.1013 |
| 0.30 | 0.30 | 0.8678 |
|  | 0.40 | 0.6314 |
|  | 0.50 | 0.4422 |
|  | 0.60 | 0.3615 |
|  | 0.70 | 0.3380 |
| 0.40 | 0.30 | 0.9375 |
|  | 0.40 | 0.8257 |
|  | 0.50 | 0.7363 |
|  | 0.60 | 0.6982 |
|  | 0.70 | 0.6871 |
| 0.50 | 0.30 | 0.9845 |
|  | 0.40 | 0.9568 |
|  | 0.50 | 0.9347 |
|  | 0.60 | 0.9252 |
|  | 0.70 | 0.9225 |

**ANALYSIS PLAN**

Demographic/clinical characteristics and safety data of the patients will be summarized using descriptive statistics such as mean, standard deviation, median and range. For the primary efficacy analysis, we will estimate the CMR (cohort 1) and OR (cohort 2) for the combination treatment, along with the 95% credible interval. For the efficacy of cohort 1, a sample size of 90 patients ensure a posterior 95% credible interval for CMR of (0.25, 0.44) if there are 31 patients achieve CMR out of 90 patients using the same prior as the monitoring rule above assuming the trial is not terminated early, under the assumption of a 35% CMR. For the efficacy of cohort 2, a sample size of 30 patients ensure a posterior 95% credible interval for OR of (0.36, 0.64), if the trial is not terminated early, under the assumption of a 50% OR. Patients who drop out of the study before completing all the cycles will be treated as “failures” for the primary analysis. The association between response and patient’s clinical characteristics will be examined by Wilcoxon’s rank sum test or Fisher’s exact test, as appropriate. Toxicity type, severity and

attribution will be summarized for each patient using frequency tables. The distribution of time-to-event endpoints (EFS and OS) will be estimated using the method of Kaplan and Meier. Comparisons of time- to-event endpoints by important subgroups will be made using the log-rank tests.

The Investigator is responsible for completing an efficacy/safety summary report and submitting it to the IND office Medical Affairs and Safety Group for review. This should be submitted after the first 5 evaluable patients per cohort, complete 3 cycles of study treatment, and every 5 evaluable patients per cohort, thereafter.

1. **Data Management and Confidentiality**

**Data Collection and Management Responsibilities**

Data Capture: Data will be entered in the MD Anderson institutionally approved database (s) Electronic Data Capture Tool, a CFR 21 Part 11 compliant system. All eligibility criteria must be documented prior to treatment initiation**.**

All data collected will be used only for research purposes. Identifiers (name, medical record number, date of birth, treatment diagnosis, imaging tests, follow-up and death) may be collected but will be replaced by study numbers in the analytic files. Patient identifiers will be confidentially collected and securely maintained on a password protected server located behind the institutional firewall. Access to identifiers will follow IRB and MDA information security rules and regulations. The master database file will be accessible only to the Principal Investigator, approved collaborators, and research staff designated on the delegation of authority log.

**Accuracy of Data Collection:** The MD Anderson Principal Investigator will be the final arbiter of response and toxicity, should a difference of opinion exist.

**Confidentiality**

All data and records generated during this study will be kept confidential in accordance with Institutional policies and HIPAA on subject privacy and that the Investigator and other site personnel will not use such data and records for any purpose other than conducting the study.

**Genomic Data Sharing Plan**

Not applicable

**Incidental/Secondary Findings Disclosure Procedure**

Not applicable

1. REFERENCES
2. Pui CH, Evans WE. Acute lymphoblastic leukemia. *N Engl J Med*. 1998;339(9):605-615.
3. Rivera GK, Raimondi SC, Hancock ML, et al. Improved outcome in childhood acute lymphoblastic leukaemia with reinforced early treatment and rotational combination chemotherapy. *Lancet*. 1991;337(8733):61-66.
4. Kantarjian HM, Walters RS, Keating MJ, et al. Results of the vincristine, doxorubicin, and dexamethasone regimen in adults with standard- and high-risk acute lymphocytic leukemia. *J Clin Oncol*. 1990;8(6):994-1004.
5. Hoelzer D, Thiel E, Loffler H, et al. Prognostic factors in a multicenter study for treatment of acute lymphoblastic leukemia in adults. *Blood*. 1988;71(1):123-131.
6. Gaynor J, Chapman D, Little C, et al. A cause-specific hazard rate analysis of prognostic factors among 199 adults with acute lymphoblastic leukemia: the Memorial Hospital experience since 1969. *J Clin Oncol*. 1988;6(6):1014-1030.
7. Hussein KK, Dahlberg S, Head D, et al. Treatment of acute lymphoblastic leukemia in adults with intensive induction, consolidation, and maintenance chemotherapy. *Blood*. 1989;73(1):57-63.
8. Larson RA, Dodge RK, Burns CP, et al. A five-drug remission induction regimen with intensive consolidation for adults with acute lymphoblastic leukemia: cancer and leukemia group B study 8811. *Blood*. 1995;85(8):2025-2037.
9. Kantarjian HM, O'Brien S, Smith TL, et al. Results of treatment with hyper-CVAD, a dose- intensive regimen, in adult acute lymphocytic leukemia. *J Clin Oncol*. 2000;18(3):547-561.
10. Kantarjian H, Thomas D, O'Brien S, et al. Long-term follow-up results of hyperfractionated cyclophosphamide, vincristine, doxorubicin, and dexamethasone (Hyper-CVAD), a dose-intensive regimen, in adult acute lymphocytic leukemia. *Cancer*. 2004;101(12):2788-2801.
11. Faderl S, Kantarjian HM, Thomas DA, et al. Outcome of Philadelphia chromosome-positive adult acute lymphoblastic leukemia. *Leuk Lymphoma*. 2000;36(3-4):263-273.
12. Ottmann OG, Druker BJ, Sawyers CL, et al. A phase 2 study of imatinib in patients with relapsed or refractory Philadelphia chromosome-positive acute lymphoid leukemias. *Blood*. 2002;100(6):1965- 1971.
13. Thomas DA, Faderl S, Cortes J, et al. Treatment of Philadelphia chromosome-positive acute lymphocytic leukemia with hyper-CVAD and imatinib mesylate. *Blood*. 2004;103(12):4396-4407.
14. Towatari M, Yanada M, Usui N, et al. Combination of intensive chemotherapy and imatinib can rapidly induce high-quality complete remission for a majority of patients with newly diagnosed BCR-ABL- positive acute lymphoblastic leukemia. *Blood*. 2004;104(12):3507-3512.
15. Ravandi F, O'Brien SM, Cortes JE, et al. Long-term follow-up of a phase 2 study of chemotherapy plus dasatinib for the initial treatment of patients with Philadelphia chromosome-positive acute lymphoblastic leukemia. *Cancer*. 2015;121(23):4158-4164.
16. O'Brien S, Thomas DA, Ravandi F, Faderl S, Pierce S, Kantarjian H. Results of the hyperfractionated cyclophosphamide, vincristine, doxorubicin, and dexamethasone regimen in elderly patients with acute lymphocytic leukemia. *Cancer*. 2008;113(8):2097-2101.
17. Ottmann OG, Wassmann B, Pfeifer H, et al. Imatinib compared with chemotherapy as front-line treatment of elderly patients with Philadelphia chromosome-positive acute lymphoblastic leukemia (Ph+ALL). *Cancer*. 2007;109(10):2068-2076.
18. Vignetti M, Fazi P, Cimino G, et al. Imatinib plus steroids induces complete remissions and prolonged survival in elderly Philadelphia chromosome-positive patients with acute lymphoblastic leukemia without additional chemotherapy: results of the Gruppo Italiano Malattie Ematologiche dell'Adulto (GIMEMA) LAL0201-B protocol. *Blood*. 2007;109(9):3676-3678.
19. Foa R, Vitale A, Vignetti M, et al. Dasatinib as first-line treatment for adult patients with Philadelphia chromosome-positive acute lymphoblastic leukemia. *Blood*. 2011;118(25):6521-6528.
20. Jabbour E, O'Brien S, Sasaki K, et al. Frontline Inotuzumab Ozogamicin in Combination with Low-Intensity Chemotherapy (mini-hyper-CVD) for Older Patients with Acute Lymphoblastic Leukemia (ALL). *Blood*. 2015;126(23):83-83.
21. Martinelli G, Dombret H, Chevallier P, et al. Complete Molecular and Hematologic Response in Adult Patients with Relapsed/Refractory (R/R) Philadelphia Chromosome-Positive B-Precursor Acute Lymphoblastic Leukemia (ALL) Following Treatment with Blinatumomab: Results from a Phase 2 Single- Arm, Multice…. *Blood*. 2015;126(23):679-679.
22. O'Hare T, Shakespeare WC, Zhu X, et al. AP24534, a pan-BCR-ABL inhibitor for chronic myeloid leukemia, potently inhibits the T315I mutant and overcomes mutation-based resistance. *Cancer Cell*. 2009;16(5):401-412.
23. Quintas-Cardama A, Cortes J. Therapeutic options against BCR-ABL1 T315I-positive chronic myelogenous leukemia. *Clin Cancer Res*. 2008;14(14):4392-4399.
24. Cortes JE, Kantarjian H, Shah NP, et al. Ponatinib in refractory Philadelphia chromosome- positive leukemias. *N Engl J Med*. 2012;367(22):2075-2088.
25. Cortes JE, Kim DW, Pinilla-Ibarz J, et al. A phase 2 trial of ponatinib in Philadelphia chromosome- positive leukemias. *N Engl J Med*. 2013;369(19):1783-1796.
26. Jabbour E, Kantarjian H, Ravandi F, et al. Combination of hyper-CVAD with ponatinib as first-line therapy for patients with Philadelphia chromosome-positive acute lymphoblastic leukaemia: a single- centre, phase 2 study. *Lancet Oncol*. 2015;16(15):1547-1555.
27. Topp MS, Kufer P, Gokbuget N, et al. Targeted therapy with the T-cell-engaging antibody blinatumomab of chemotherapy-refractory minimal residual disease in B-lineage acute lymphoblastic leukemia patients results in high response rate and prolonged leukemia-free survival. J Clin Oncol 2011; 29: 2493-98.
28. Topp MS, Gokbuget N, Zugmaier G, et al. Long-term follow-up of hematologic relapse-free survival in a phase 2 study of blinatumomab in patients with MRD in B-lineage ALL. Blood 2012; 120: 5185-5187.
29. Nagorsen D, Kufer P, Baeuerle PA, Bargou R. Blinatumomab: a historical perspective. Pharmacol Ther 2012; 136: 334-342.
30. Topp M, Gokbuget N, Zugmaier G, et al. Effect of anti-CD19 BiTE blinatumomab on complete remission rate and overall survival in adult patients with relapsed/refractory B-precursor ALL. J Clin Oncol 2012; 30: Abstract 6500.

30 Schmitt MW, Fox EJ, Prindle MJ, et al. Sequencing small genomic targets with high efficiency and extreme accuracy. Nat Methods. 2015 May;12(5):423-5.
